# Exploiting the Potential of Bioreactors for Creating Spatial Organization in the Soil Microbiome: A Strategy for Increasing Sustainable Agricultural Practices

**DOI:** 10.3390/microorganisms10071464

**Published:** 2022-07-20

**Authors:** Carlos Fernando Gutiérrez, Nicolás Rodríguez-Romero, Siobhon Egan, Elaine Holmes, Janeth Sanabria

**Affiliations:** 1 Environmental Microbiology and Biotechnology Laboratory, Engineering School of Environmental & Natural Resources, Engineering Faculty, Universidad del Valle, Meléndez Campus, Cali 76001, Colombia; carlos.f.gutierrez@correounivalle.edu.co (C.F.G.); andres.nicolas.rodriguez@correounivalle.edu.co (N.R.-R.); 2 Australian National Phenome Center, Murdoch University, Perth 6150, Australia; siobhon.egan@murdoch.edu.au (S.E.); elaine.holmes@murdoch.edu.au (E.H.)

**Keywords:** biofertilizer, plant-growth-promoting, batch, continuous culture, diazotrophic bacteria

## Abstract

Industrial production of synthetic nitrogen fertilizers and their crop application have caused considerable environmental impacts. Some eco-friendly alternatives try to solve them but raise some restrictions. We tested a novel method to produce a nitrogen bioinoculant by enriching a soil microbial community in bioreactors supplying N_2_ by air pumping. The biomass enriched with diazotrophic bacteria was diluted and applied to N-depleted and sterilized soil of tomato plants. We estimated microbial composition and diversity by 16S rRNA metabarcoding from soil and bioreactors at different run times and during plant uprooting. Bioreactors promoted the N-fixing microbial community and revealed a hided diversity. One hundred twenty-four (124) operational taxonomic units (OTUs) were assigned to bacteria with a greater Shannon diversity during the reactor’s steady state. A total of 753 OTUs were found in the rhizospheres with higher biodiversity when the lowest concentration of bacteria was applied. The apparent bacterial abundance in the batch and continuous bioreactors suggested a more specific functional ecological organization. We demonstrate the usefulness of bioreactors to evidence hidden diversity in the soil when it passes through bioreactors. By obtaining the same growth of inoculated plants and the control with chemical synthesis fertilizers, we evidence the potential of the methodology that we have called directed bioprospecting to grow a complex nitrogen-fixing microbial community. The simplicity of the reactor’s operation makes its application promising for developing countries with low technological progress.

## 1. Introduction

The intensification of agriculture through methods including intensive fertilization has substantially increased food availability but has imposed severe environmental consequences. Nitrogen-based synthetic fertilizer production through the Haber–Bosch process consumes more than 1% of the world’s total energy generated, using about 2% of the natural gas extracted and emitting more than 300 million metric tons of carbon dioxide [1]. In addition, applying N-based fertilizers to crops has led to a global nitrogen cycle disturbance, illustrated by the increasing eutrophication of land and water bodies, greenhouse gas emissions, and biodiversity losses, particularly of soil microbial communities [2,3]. In 2050, the world’s population is predicted to increase to around 10 billion, with consequent increases in the demand for food and agricultural expansion [4,5]. Some ecological alternatives involve the intensive use of crops that replace soil nitrogen content, such as legume-based fertilization systems, organic farming, and microbial bio-inoculants, using endophytic microbes to increase the supply of nutrients to crops. However, not all legumes are liable to intercrop with nonleguminous plants; the rotation process can take longer than expected or defy large-scale application [6]. Using manure, compost, and plant residues may lead to the accumulation of pharmaceuticals and antibiotics [7,8]. Moreover, it is insufficient to supply the demand for crop production [9]. As a result, abandoning synthetic N-fertilizers could lead to nutrient undersupply, even with increased legume cropping.

Regarding bio-inoculants, even though their world market is growing, their application is still marginal compared with chemical fertilizers [10]. Several factors hinder their use, including identifying and tracking inoculated strains in the field, the poor understanding of relationships between microorganisms and plants, and complex production technology [11]. Plants and microbial communities co-exist depending on their mutual species interactions, the microenvironment generated by the physicochemical conditions of the soil, and the ability to adapt to changes in each of these conditions quickly. Therefore, the effects of plant species on microbial taxa are often not easy to predict a priori [12]. Even within a single species, plants can select different subsets of microorganisms at different stages of development, presumably relating to specific functions [13]. Thus, there is not a universally applicable bioinoculant for all crop types and soils. Manipulating active microbial communities in agriculture and developing new microbiome engineering approaches to address these challenges is a priority. These microbiome engineering approaches allow the manipulation and study of microbial communities in situ, without isolating species or model communities in the laboratory [14], and promote plant fitness and health [15]. Specifically, integrating microbiome engineering theory with bioprocess engineering offers a top-down approach. The sample is a blank canvas, and external stimuli are applied to identify patterns and self-assembled construction forms associated with specific conditions [16]. This approach has been used to develop strategies for enhancing bioremediation, providing additional nitrogen, and identifying adaptation strategies associated with nitrogen fixer communities [17,18]. The present study aimed to engineer a diazotrophic community in reactors using the self-assembly approach. Subsequently, the community was introduced into the rhizosphere of a nonlegume plant, and its capacity as a growth promoter was assessed. There was no difference in growth parameters between plants grown in chemical fertilizer versus inoculation, which suggests that this methodology could be used to replace chemical fertilization.

## 2. Materials and Methods

### 2.1. Microbial Inoculum Pre-Adaptation in Batch Reactors

A total of 5 kg of bulk soil, consisting of hydrogenous clastic surface deposits with a clay loam texture, with a taxonomy corresponding to Entic Haplustolls [19], was collected (20 cm depth) from a forest aged >30 years in a naturally restored process, located next to the Experimental Station at the campus of Universidad del Valle (3°22′23″ N, 76°31′51″ W; Cali, Colombia). The soil was dried by air and sieved (<2 mm) to remove root fragments and debris before measurement of physicochemical parameters. Colorimetric methods were used to measure total phosphorus (TP) and total organic carbon (TOC) content. Total Kjeldahl Nitrogen (TKN) concentration was determined by Kjeldahl digestion of an unfiltered sample according to the APHA method [20]. The pH was measured in distilled water at a 1:4 soil-to-water volume ratio with a glass electrode. All analyses were carried out in triplicate. The soil had a pH of 6.09, total organic carbon of 29.71 g kg^−1^ soil, TKN of 3.045 g kg^−1^ soil, and TP of 55.73 mg kg^−1^ soil.

**Figure 1:**
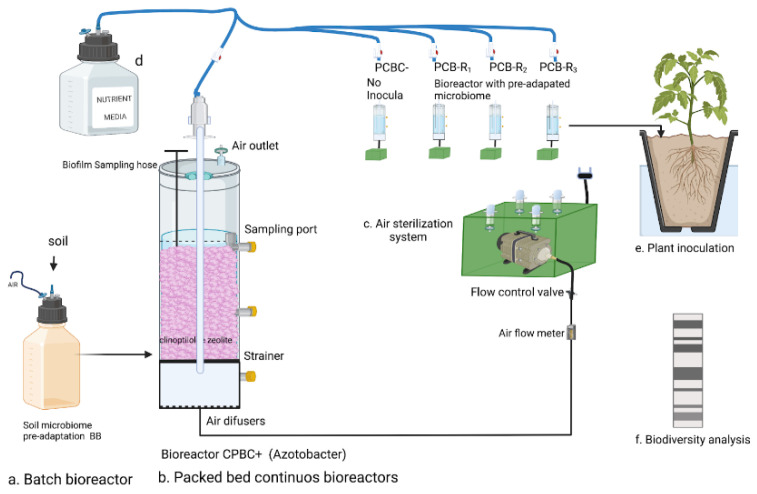
Experimental setup. (**a**) Batch bioreactor (BB) was fed with glucose-citrate as the only carbon source and N-depleted; (**b**) set of continuously packed bioreactors (PcB-R1 to PcB-R3 and PcB-C+), fed with citrate as the only carbon source and N-depleted; (**c**) sterile box provided with Nylon Syringe Filter, 0.22 µm and pump. The sterile air reaches the bioreactors through a plastic conduit. (**d**) Media supply; (**e**) pot model for plant inoculation; (**f**) 16srARN was used for microbial analysis.

After homogenization, one gram of the same soil was pulverized and diluted in 2.0 L of sterilized, nitrogen-free, RBA solution (DSMZ—Medium 441) and glucose (8.0 g L^−1^) as the only carbon source. The soil slurry was continuously mixed with a stir plate set at 100 rpm and maintained in the dark in a batch-type bioreactor where the content was continuously mixed (BB) (Figure 1a). The bottle cap was connected to a 0.2 mm microfilter to allow oxygen exchange while avoiding airborne microbial contamination. Two days after commencing the stirring process, the nutrient solution was switched to the sterilized, N-free modified Hoagland solution [21]: (g L^−1^), 7.5 of KCl, 5.55 of CaCl_2_, 2.0 of KH_2_PO_4_, 2.0 of MgSO_4_, 1.5 of FeSO_4_, and 1 mL of trace element solution SL-6 (DSMZ medium 27). A solution of 80% glucose and 20% citrate (principal root exudates of some nonlegume plants [22] (*w*/*w* = 6.4 g/1.7 g, respectively) was used as the carbon source. Every two days, the citrate was increased by 20% until reaching 100% (8.5 g). After ten days of adaptation, 120 mL of the microbial community suspension (SMC) was transferred to the packed-bed bioreactors (PcB). A volume of 850 mL was centrifuged for 60 min at 9000× *g*, and the pellets were stored in ethanol (70%) at −20 °C for DNA extraction. The Chemical Oxygen Demand (COD) was measured at 1200 mg L^−1^ and pH 5.5.

### 2.2. Packed-Bed Bioreactor’s Configuration

Bioreactor vessels were built using a sterile acrylic tube (0.42 m high, 10.16 cm diameter) containing sterilized clinoptilolite zeolite as the packed material (4 mm diameter) and five biologically aerated packed-bed bioreactors (PcBs). The total reactor volume was 1.8 L with a working volume of 1.2 L (Figure 1). Three reactors were used as experimental replicates (PcB-R1, PcB-R2, and PcB-R3). The fourth bioreactor served as a negative control (PcB-C−) without inoculation, and the last one was used as a positive control (PcB-C+) inoculated with 120 mL of the well-known and applied inoculant, *Azotobacter vinelandii*, obtained from the DSMZ (Braunschweig, Germany DSM 2290), which was reactivated in a 2.0 L sterilized RBA solution (DSMZ medium 441). All reactors were fed by drip gravity and operated continuously with a hydraulic retention time (HRT) of 7 days. The airflow was microfiltered in each reactor to avoid microbial contamination, and an air pump maintained at a rate of 4 mL/min was used at room temperature (22 ± 2 °C).

### 2.3. Reactor Sampling and Processing

We took 50 mL of unfiltered samples from each reactor every two weeks. TKN, ammonium (NH_4_^+^), nitrites (NO_2_^−^), nitrates (NO_3_^−^), and Chemical Oxygen Demand (COD) assays were performed. The physicochemical analysis was performed according to standard methods [23]. The microbial biomass was calculated based on the weight of the pellet after centrifugation of 100 mL at 13,000× *g* for 20 min (Thermo Scientific Heraeus Multifuge centrifuge X1, Walthman, MA, USA). Optical density (OD_600_) was measured using a vision spectrophotometer SP-2001SD manufactured by Hoefer, Inc. Holliston, MA, USA).

### 2.4. Fertilizer’s Solution Preparation

The microbial solution was obtained from a composite sample of the effluents from the three reactors, PcB-R1, PcB-R2, and PcB-R3 effluent. An amount of 600 mL of effluent was collected in a sterile bottle on the day of application. The biomass collected was centrifuged at 13,000× *g* for 20 min and resuspended in the N-free modified Hoagland solution to normalize the microbial concentration. Treatments consisted of the N-free modified Hoagland solution supplied with a microbial solution adjusted to different optical densities values: 0.1 (P-O.1OD), 0.2 (P-O.2OD), and 0.3 (P-O.1OD). The positive control (P-C+) consisted of a Hoagland solution supplied with (g/L) 2 M KNO_3_, 202; 2.5 of 2 M Ca(NO_3_)2•4H_2_O, 236. For the negative control (P-C-), sterilized deionized water (SDW) was used. Each treatment had four replicates that were randomly distributed on the germination tray. Every two days, the tray cell received a volume of 200 μL of the microbial solution, Hoagland media, or water. The dose of microbial community was doubled every week as the plants grew. Soil moisture was maintained by watering with SDW when necessary.

### 2.5. Soil and Seed Selection for Planting

From the same soil previously described, 50 kg of mixed sample was gently air-dried, sieved (2 mm), placed on ten tin trays (5 × 25 × 50 cm), wrapped with kraft foil, autoclaved (120 °C—2 h), and dried in an oven (50 °C—4 h). Wild tomato (*Solanum pimpinellifolium*) fruits were purchased from a local market. The seeds were extracted, washed, and dried at room temperature (22 °C) for two days in the laboratory. The seeds were then surface-sterilized by gently shaking with 70% ethanol (2 min), followed by 2.5% sodium hypochlorite solution (5 min), and five rinses in sterile distilled water (SDW). They were then soaked in SDW in sterilized Petri dishes and allowed to germinate in the dark. After three days, germination seeds were transferred to sterilized 6 × 12 cells filled with sterile forest soil.

### 2.6. Tomato Plant Growth Assessment

Once a week, the plant stem length, the root length, the root width, the root volume, and the total plant length were measured. On the seventh week, seven random plants per treatment were carefully uprooted. The remaining plants were carefully transferred to a garden pot (15 × 30 cm, diameter × height), filled with 1.5 kg of sterilized soil, and grown under greenhouse conditions (medium temperature: 24.5 °C, natural light photoperiod 12 h, and 72% relative humidity). The fertilizer dosing scheme continued with 1.2 mL three times per week and increased to 400 μL weekly as the plants grew. The final harvest of four plants was made after 24 weeks.

### 2.7. DNA Extraction, Sequencing, and Metabarcoding Analysis

**Figure 2:**
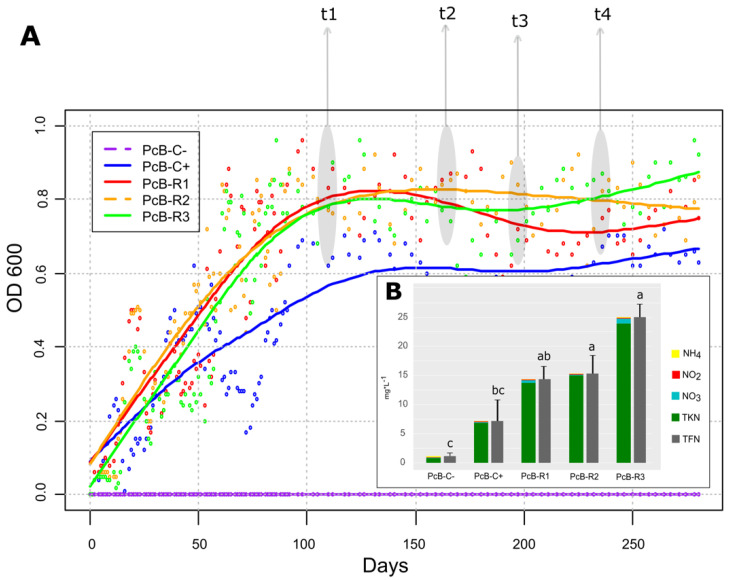
(**A**) Growth curve of the soil-adapted microbiome and *Azotobacter vinelandii* in the packed continuous bioreactors. (**B**) Nitrogen compounds accumulated in the bioreactors at the end of the experiment. TFN: Total fixed nitrogen; NH_4_: ammonium; NO_2_: nitrite; NO_3_: nitrate; TKN: total Kjeldahl nitrogen. Different letters show statistically significant differences (Tukey *p* < 0.05). (t1, t2, t3, t4): moments of sampling for DNA extractions and metabarcoding analysis.

Nine samples were taken for molecular analysis: one from bare soil, one from the Batch Bioreactor (BB), four from Packed Bioreactors (PcBs) (at different times during steady state), and three from the rhizosphere (at the end of each treatment). Total DNA was extracted from 250 mg of initial bulk and rhizosphere soil at the end of the greenhouse experiments. Rhizosphere soil was obtained after treatments by shaking each plant by hand to remove large soil aggregates and loosely adhering soil. The soil remaining on the roots was collected using the protocol from [24]. For the Batch reactor DNA extraction, 850 mL of the effluent was collected after ten days of operation and centrifuged for 60 min at 9000× *g*, and the pellet was resuspended in 0.8 mL of PBS. For packed bioreactors, we collected a composite sample of the three repetitions of bioreactors at different times of the stationary state of the growth curve: PcB_t1_ = 112, PcB_t2_ = 160, PcB_t3_ = 200, and PcB_t4_ = 240 days, as shown in Figure 2. All extractions were performed using a NucleoSpin^®^ soil genomic DNA extraction kit (Machery-Nagel, Dueren Germany) using SL1 lysis buffer and 100 μL of enhancer SX. The lysis step was repeated three times for each sample. After the lysis step, extractions were performed following the manufacturer’s standard protocols. The DNA was sent to MrDNA laboratories (MrDNA, Shallowater, TX, USA). DNA yield and purity were measured using a micro-volume fluorospectrometer (Thermo Scientific, NanoDrop Technologies, Wilmington, DE, USA). The 16S rRNA gene amplicon (341-785 v3-v4 region) Illumina sequencing was processed using Qiime2, version 2019.1 [25]. Denoising, quality filtering, and chimera checking (‘consensus’) were performed by the ‘dada2’ plugin [26], and taxonomy was assigned against the SILVA database (138.1 release; 2020).

### 2.8. Statistical Analysis

The results were subjected to an analysis of variance (*p* < 0.05) and a subsequent post hoc Tukey test (*p* < 0.05). For each of the cases, the assumptions of normality were tested using the Shapiro–Wilk test (*p* < 0.05) and the homogeneity of variances using the Bartlett test (*p* < 0.05). All analyses were performed using R version 3.5 [27].

## 3. Results

### 3.1. Reactor’s Startup and Performance

The growth curve of the soil microbial community in the bioreactor (PcB-R1, PcB-R2, and PcB-R3) was similar, reaching the steady-state after 100 days of operation (Figure 2A). The PcB-C+ reached a steady-state in approximately 120 days. All reactors achieved the highest optical density (OD) and biomass values at 197 days. The OD in the reactor was 0.63, 0.75, 0.86, and 0.921 (PcB-C+, PcB-R1, PcB-R2, and PcB-R3, respectively). The biomass increased from undetectable to 865, 1230, 1452, and 2147 mg/L in PcB-C+, PcB-R1, PcB-R2, and PcB-R3, respectively. The COD and N-values indicated that both *Azotobacter vinelandii* pure culture and the Soil-Adapted Microbiome (SAM) consumed the citric acid present in the nutrient media and fixed atmospheric N_2_ pumped into the bioreactors. The negative control reactor (PcB-C−) did not have any OD or biomass increase during the entire operation. The nitrogen fixation was calculated as TFN = TKN + NH_4_ + NO_2_ + NO_3_. At the end of the bioreactor’s performance, an increase in NO_3_ was observed in the effluent of the PcBs. All the results of the physicochemical characterization are shown in Supplementary Materials File S1, Tables S1–S6.

### 3.2. Bioinoculant Characteristics

At the moment of the first inoculation of the tomato plants, the PcB had reached an average N_fixed_ of 18.24 (±5.89) mg/L. The biomass was 1609.7 (±478.4) mg/L and had an OD_600_ of 0.67 (±0.19). During the tomato fertilization process, the PcBs maintained an average OD of 0.78 (±0.09) without significant concentration changes until the final harvest of the tomato plants at 280 days of reactor operation.

### 3.3. Composition of the Bacterial Community in Bioreactors

**Figure 3:**
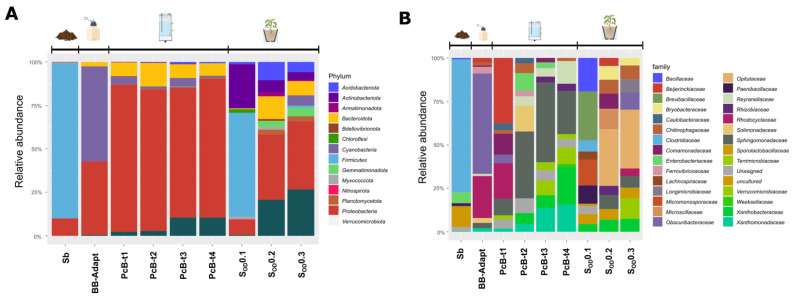
Complete progressive microbial changes in the relative abundance of bacteria along with the experiment: (**A**) Phylum and (**B**) Family. Sb: Bare soil; BB: batch bioreactor; PcB: Packed Bioreactor; S_OD_: Rhizospheric soil after application of microbial solution; numbers mean bacterial OD concentration.

The soil microbiome drastically changed during the adaptation process in the batch bioreactor (BB), as well as in the packed continuous bioreactors (PcBs). The soil microbiomes diversified from four initial phyla (Firmicutes 89.8%, Proteobacteria 9.8%, Actinobacteria 0.3%, and Bacteriodota 0.1%) to eight (Proteobacteria 84.9%, Bacteroidota 7.8%, Cyanobacteria 4.5%, Actinobacteria 0.3%, Planctomycetota 0.2%, Bdellovibrionota 0.2%, and Firmicutes 0.1%) in the BB. In the PcBs at different times, we found: Proteobacteria 42.5, 81.4, 74.9, and 79.7%; Bacteroidota 2.6, 13.6, 8.1, and 7.1%; Verrucomicrobia 0.3, 2.7, 10.3, and 10.4; Cyanobacteria 54.5, 1.2, 4.9, and 1.0%; Firmicutes 0.0, 0.0, 0.2, and 0.2%; Acidobacteria 0.0, 0.3, 1.0, and 0.7%; Planctomycetes 0.0, 0.5, 0.6, and 0.7%. Changes in all phyla and families are shown in Figure 3.

In terms of the genus relative abundance, the most notable changes when comparing soil with the batch bioreactor (S-BB) were: *Beijerinckia* (0 to 33.4%), *Zoogloea* (0 to 18.1%), *Novosphingobium* (0.1 to 7.4%), *Pelomonas* (0 to 10.1%), c Bacteroidia (0 to 4.6%), *Candidatus_Obscuribacter* (0 to 4.4%), and *Phenylobacterium*, (from 0 to 3%).

Meanwhile, the following genera diminished (Soil-BB): *Clostridium_sensu_stricto_12* (68.2 to 0.1%), *Sporolactobacillus* (11.5 to 0.1%), *Clostridium_sensu_stricto _1* (5.5 to 0%), *Paenibacillus* (1.4 to 0%), and *Bacillus* (0.8 to 0%). The following shows the comparison of the growth in the batch reactor with the early stages in the packed bioreactors (BB-PcB_t1_): *Candidatus Obscuribacter* (4.4 to 54.3%), *Zoogloea* (18.1 to 22.8%), *Ferrovibrio* (0 to 3.6%), and *Hydrocarboniphaga* (0 to 2.7%). In contrast, the relative abundance of *Terrimonas* (1.8 to 1%), *Terrimicrobium* (2 to 0.3%), *Phenylobacterium* (3 to 0.1%)*,* cBacteroidia_783535a03c2e41866d160d349546fd1b (4.6 to 0.2%), *Novosphingobium* (7.4 to 1.1%)*, Pelomonas* (10.1 to 0.1%)*,* and *Beijerinckia* (33.4 to 2.5%) decreased from BB to PcB_t1_. At the end of the growth period in the packed bioreactors (PcB_t1_ to PcB_t4_), the relative abundance decreased in *Candidatus_Obscuribacter* (54.3 to 0.1), *Zoogloea* (22.8 to 0%), and *Ferrovibrio* (3.6 to 0.5%), while *Xanthobacter* (0.3 to 17.9%) Sphingobium (0.8 to 17.5%), *Pseudoxanthomonas* (1.7 to 12.2%), *Reyranella* (1.1 to 10.8%), *Prosthecobacter* (0 to 7.9%), c Bacteroidia_783535a03c2e41866d160d349546fd1b (0.2 to 3.6%), and *Pseudomonas* (0 to 2.3%) increased.

### 3.4. Composition of the Bacterial Community in the Soil

**Figure 4:**
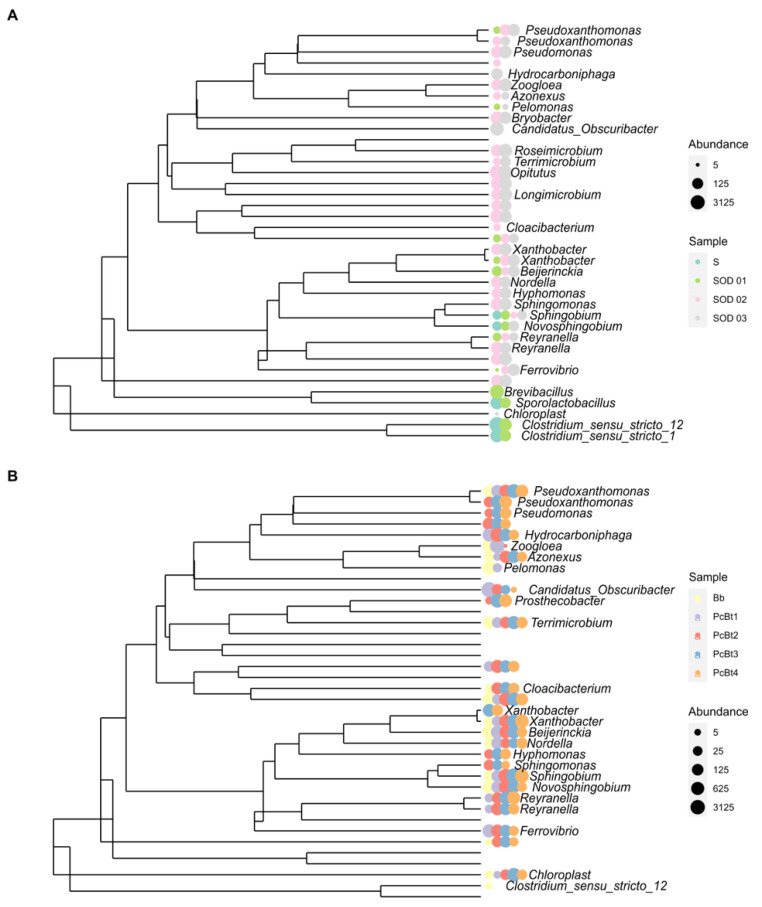
Evolution of the main identified genus along the adaptation process. (**A**) S: Bare Soil without treatment. S_OD_: Rhizospheric soil after application of microbial solution, where numbers mean bacterial OD concentration. (**B**) Bb: Batch reactor (complete mix, glucose to citrate finding, and N_2_ by air supply) after ten days. PcB: Packed Bioreactors (citrate as the only carbon source and N_2_ by air supply) (PcB_t1_ = 112, PcB_t2_ = 160, PcB_t3_ = 200, and PcB_t4_ = 240 days). The size of the circles represents rRNA-16s abundance.

The soil underwent a considerable change in microbial biodiversity after applications of the enriched microbiome. The phyla Firmicutes 89.8%, Proteobacteria 9.8%, and Actinobacteria 0.3% were observed in soil without treatment. The most abundant genera are the sporulated Clostridia, Sporolactobacillus, and the facultative anaerobic *Klebsiella.* After treatments with the packed bioreactors’ microbial inoculation in the rhizosphere, the most abundant genus was *Opitutus*. Comparing the relative abundance between the soil before the inoculation and after sterilization and re-inoculation (bare soil–rhizospheric soil), the most important changes were observed for *Opitutus* (0 to 18.3%), *Roseimicrobium* (1 to 6.2%), *Candidatus_Obscuribacter* (2 to 5.5%), Longimicrobium (3 to 4.1%), *Rhodospirillales* (4 to 2.6%), *Zoogloea* (5 to 2.5%), *Xanthobacter* (6 to 2.4%), and *Pseudomonas* (7 to 2.4%) increasing. *Clostridium_sensu_stricto_12*, *Sporolactobacillus*, and *Clostridium_sensu_stricto_1* identified in the initial soil sample were not identified in treated soils. Moreover, the genera *Azonexus*, *Cloacibacterium*, and *Pelomonas,* and an unassigned group of the family Enterobacteriaceae, were identified in bioreactors but were not subsequently present in the soils. The evolution of bacterial genera through the treatments is shown in Figure 4.

The Shannon diversity was lower in the initial soil but increased during the acclimatization process in BB. Through the steady-state, it varied from 2.0 (PcBt1), 3.30 (PcBt2), 3.30 (PcBt3), and 3.27 (PcBt4). The Simpson index showed that alpha diversity was lowest in the soil initial soil (0.6) and increased in bioreactors, reaching higher levels during the stationary state from PcBt1 to PcBt4 (0.70, 0.975, 0.875, and 0.974, respectively). The Chao1 index showed lower diversity in the soil sample (low index: 40) and higher diversity in the acclimation and states PcBt1-PcBt4n (81, 98, 122, and 81, respectively). In the Supplementary Materials File S2 phyloseq plots, in Figures S3 and S4, the biodiversity index is presented and the community dynamic using heat maps is presented in Figures S5–S7.

### 3.5. The Phenotypic Response of the Tomato Plants

**Figure 5:**
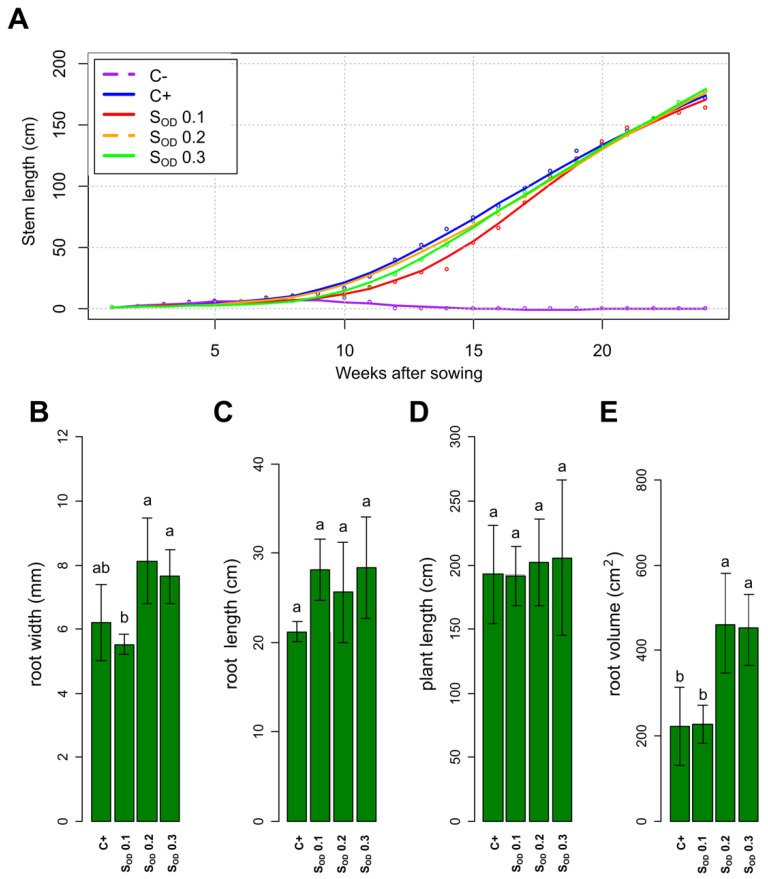
Growth parameters of tomato plants in the greenhouse experiment. (**A**) Stem length from week one to week 24 after sowing, C−: plants died at week seven due to the absence of mineral nutrients in the soil (purple line). (**B**) Root width at week 24 after sowing. (**C**) Root length at week 24 after sowing. (**D**) Plant length at week 24 after sowing. (**E**) Root volume at week 24 after sowing. Different letters show statistically significant differences (Tukey *p* < 0.05).

In the final greenhouse experiment (week 24 after sowing), the growth of tomato plants C+ and plants inoculated with reactor effluent (S_OD_ 0.1, S_OD_ 0.2, and S_OD_ 0.3) did not present statistical differences (*p* < 0.05) for stem length, root width, root length, and plant length (Figure 5A–D, respectively). However, there were differences in root volume (Figure 5E). These results suggest that with the nitrogen supplied by the microorganisms, the same growth is obtained as with the added ammoniacal nitrogen.

## 4. Discussion

### 4.1. Bioreactors and Microbial Biodiversity

The results indicate that the microbial community in bioreactors was capable of fixing nitrogen when provided with citric acid as the only carbon source. Different bacterial species carried out nitrogen fixation, and the nitrogen-fixing genera evolved with the growth of the plants.

**Figure 6:**
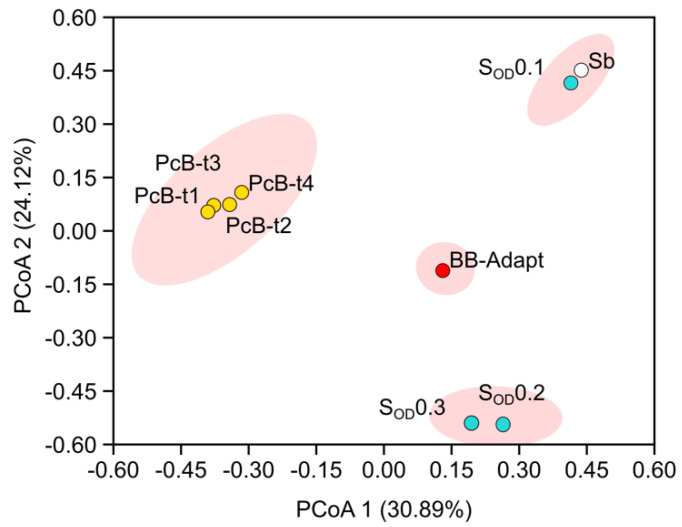
Principal coordinate analysis using the Bray–Curtis phylogenetic distance matrix based on the ASV table. Sb: Bare Soil without treatment. S_OD_: Rhizospheric soil after application of microbial solution; numbers mean bacterial OD concentration; Bb: Batch bioreactor (complete mix, glucose to citrate finding, and N_2_ by air supply) after ten days. PcB: Packed Bioreactors (citrate as the only carbon source and N_2_ by air supply) (PcB_t1 = 112, PcB_t2 = 160, PcB_t3 = 200, and PcB_t4 = 240 days).

The use of bioreactors to detect and study complex microbial communities has been demonstrated for bacteria from the human microbiome and many environmental systems [28,29]. The bioreactor strongly impacted the community structure of the soil microbiome, as shown in Figure 6 of principal components. After seeding each bioreactor with the original soil solution, the bioreactor was isolated from the surrounding environment, and media and air, which were introduced to support the microbial community, were sterilized to prevent the introduction of new micro-organisms. Therefore, all the species detected in the different stages of adaptation were present in the initial soil sample. The changes in community structure reflect the dependency on co-metabolism and nutrient transformations in a complex ecological structure. Thus, one benefit of using bioreactors is that the relative abundance of species that are initially present in low quantities and which may go undetected can be magnified, allowing the further study of their metabolic capabilities and role in the community.

We found at least three genera, *Candidatus finniella*, *Azonexus*, and *Cloacibacterium*, which were only identified in the bioreactors but not in the soil. *Candidatus finniella* has been described as an obligate intracellular parasite, an amoeboflagellate that perforates the cell walls of freshwater green algae and feeds on the algal cell contents by phagocytosis [30]. The presence of this organism means that the host protozoa are present and benefit from the bioreactors to grow. However, we do not know their role in the consortium, because we did not measure the eucaryotes; it could also mean that other protozoa are present. Protozoa are natural predators of bacteria, the species that prey on nitrogen-fixers in the soil have been studied, and their impact has been evaluated [31,32]. Therefore, it is important to control the growth of protozoa in bioreactors. In other experiments, we have achieved significant results by limiting oxygen. In the present work, a decrease in *Candidatus finniella* was observed in packed bioreactors (1.12%, 0.41%, 0.91%, and 0.27% in PcB_t1_ to PcB_r4_, respectively). *Azonexus*, a genus that has been identified as a critical endophyte for nonlegumes, occurs only under microaerobic conditions and in the absence of high concentrations of other nitrogen sources [33]. The presence of this species refers to how beneficial microorganisms can be enriched in bioreactors with potential application to agricultural practice. The third genus detected only in the bioreactors was *Cloacibacterium*, a heterotrophic bacteria identified in wastewater treatment that plays an essential role in organic matter fermentation [34]. Therefore, its presence in the bioreactors is unsurprising as fermentative bacteria are highly favored in bioreactor environments.

We analyzed the genus in the bioreactor involved with the nitrogen cycle as described by [35]. As we did not amplify the gene, we used the cross-matched names of the Uniprot database retrieved list (February 2022) and the assignment obtained from Silva2020. As expected, with no combined nitrogen input to the medium, the nitrogen fixers adapted to the growing conditions in the bioreactors determined the microbial succession. Importantly, different groups of nitrogen fixers were observed at high and low densities, demonstrating their importance in shaping ecology throughout diversity. *Zooglea* and *Beijerinkia*, in the batch reactor and the first days of adaptation in the packed reactors, at the end of the growth in the packed bioreactors, were replaced by *Sphingobim* and *Xhantobacter.* Other organisms not dependent on inorganic nitrogen also evolve differently in each type of bioreactor. The physical structure of the bioreactor environment significantly impacts the composition and, therefore, functionality of the bacterial community. The zeolite particles in the packed bioreactor provide a greater surface area for biofilms and more opportunities for localized communities.

**Figure 7:**
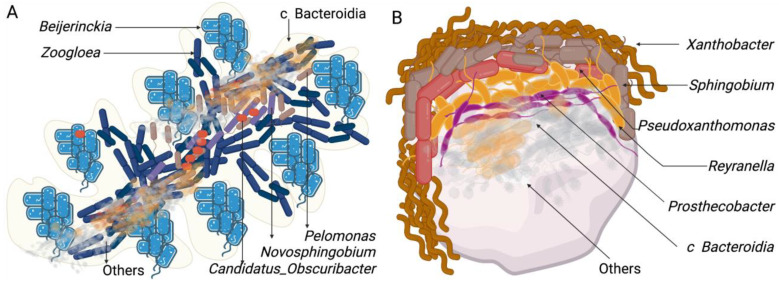
Proposed microbial organization scheme: (**A**) For flocs in batch bioreactors. (**B**) In the biofilms in the packed bioreactors after 240 days of growth.

Moreover, the environment provided by the packed bioreactor is closer to that of the natural soil environment, thereby increasing the relevance of in vitro studies. For example, *Candidatus obscurbacter* was abundant in the batch bioreactor, whose physical and possibly functional space was occupied by an unknown group of the Class Bacteroidea. Our analysis also suggests a cluster organization of fixers and nonfixers (the complete database for the study is provided in the Supplementary Materials File S3). The possible flocs in batch reactors and biofilms in the packed reactors are schematized in Figure 7.

Zooglea was favored only in the batch reactor and has stood out for its ability to form flocs in activated sludge systems and flooded soils for rice cultivation [36]. One study found that aerobiosis favored Zooglea fluctuation because of the high affinity with oxygen [37]. The genus *Beijerinckia* is also found in environments rich in oxygen and carbohydrates, a positive environment for its growth [38]. On the other hand, *Pelomonas* has been identified in fluids for hemodialysis. Its ability to grow in oligotrophic media has been demonstrated [39], while *Candidatus obscuribacter* is a facultative anaerobe that could occupy the interior of the floc [40]. These genera found in relatively high density only in BB, with diverse metabolic characteristics, could suggest the organization of co-metabolism in the floc structure facilitated by *Zooglea*. Although it was impossible to measure dissolved oxygen in the packed reactors’ microenvironment, it is assumed that less oxygen reaches the microenvironments between the zeolite particles in the bioreactor. This condition, together with the carbon source change (glucose was gradually replaced by citrate in BB, while only citrate was used in the packed bioreactors), was a determinant of the growth of the genera *Sphingomona*, *Xanthobacter*, and *Reyranella* that exhibit the ability to use in N-deficient environments and organic substrates ranging from acetate to aromatic hydrocarbons [41,42].

Proteobacteria generally prefer environments rich in labile—easily transformed—carbon sources [43] and are found to fix nitrogen in bioreactors treating wastewater with a high carbon-to-nitrogen ratio [44]. Some aquatic Verrucomicrobia (Spartobacteria and Opitutae classes with aerobic and heterotrophic metabolism) can fix nitrogen. However, they are the most abundant taxa in some natural wetlands where the organic matter and nutrient contents are significantly higher [45]. This suggests that, in BB reactors, the phyla abundances were highly affected by the nutrient ratio.

### 4.2. Rhizosphere Soil Microbiome and Plant Growth Performance

We found that plants inoculated with packed bioreactor effluent had the same growth as C+ plants that were fertilized with Hoagland solution. Plants in C− treatment died at seven weeks due to the absence of any nutrient addition, meaning that plant growth in plants in P 0.1_OD_, P 0.2_OD_, and P 0.3_OD_ was enhanced by microbial inoculation.

The soil used to grow the tomato plant was characterized by scarce nutrients, low humidity, without surface vegetation, and little contribution of organic matter. These characteristics are hostile to many groups of microorganisms and generate two parallel scenarios: one in which microorganisms remain in latent forms through resistance structures such as spores (Firmicutes 89.8%). Other groups remain metabolically active but in low abundance due to environmental and nutritional limitations (Proteobacteria 9.8%) (Figure 3a). In a recent study, it was found that the Actinobacteria (0.3%), encode a full set of CAZymes, nitrogenases, and antibiotic synthetases and is related with infertile soils [46].

A similar microbial community between the initial soil and the soil inoculated with a low inoculum density (P_0.1_OD_) suggests that the plant initially recruits a microbiome in the soil that remained viable after sterilization and colonized the rhizosphere faster compared to the taxa present in the effluent from packed bioreactors at 0.1 of optical density P_0.2_OD_. A greater abundance of genera such as *Opitutus* was observed in the rhizosphere with treatments S_OD_ 0.2 and S_OD_ 0.3. Phenotypic changes were also observed in plants roots; there was an increase in the root width and root volume as the amount of inoculum increased (Figure 5B,E); as there is a more significant microbial population, it possibly induces the plant to facilitate a more significant translocation of compounds toward the root to maintain the sizeable microbial community. However, this does not negatively affect the plant growth in stem length, meaning that an enhancement in Rubisco enzyme activity possibly causes a more efficient photosynthetic machinery due to higher CO_2_ concentrations emitted by a high rate of soil microbial respiration.

Many reports show the root phenotypic changes in response to microbial inoculations, especially under limiting environmental and sparse nutritional conditions [47,48,49]. For microbial community assessment, the space is documented as a critical factor. Reduced root diameter means less surface area for bacterial colonization for an individual root. The plant increases the surface area of its roots to recruit more microbes [49], which results in better long-term metabolic conditions for growth.

## 5. Conclusions

During the different experimental stages, the soil microbial community composition and prevalent families reflected the differences in the substrate state (dry-soil, liquid, and biofilm) and mineral and carbon sources. The reactors fed with a modified Hoagland solution with low-cost N supplied only by air and citrate as the carbon source allowed the growth of a complex nitrogen-fixing microbial community. Nevertheless, the process of reaching a steady state lasted around 80 days. NH_4_, NO_2_, and NO_3_ concentrations in the effluent were negligible, and TKN corresponded to the microbial biomass. The different communities’ compositions and their metabolic characteristics revealed the bioreactors configuration effect (soil-organic particles, batch-flocs, and packed-biofilm). The acclimatization process in the batch complete mix bioreactor selected a ‘free-swimmer’ community dominated by *Beijerinkia* and *Zooglea*. The microbial community in the biofilm from the nitrogen-fixing reactors was more diverse than those from soil or the acclimatization process and was dominated by *Xanthobacter* and *Sphingobium*. The microbial composition suggests the participation of the complete nitrogen transformations in the bioreactors. In addition, a complex nitrogen-fixing microbial community was identified in the plant root rhizospheres in each treatment. The COVID-19 pandemic and the war in Ukraine have highlighted the difficulties, especially for emerging countries with an agricultural base to access fertilizers, which, together with the environmental problems caused by their industrial production, urge local solutions to countries. Bioreactors have proved their value for enriching nitrogen-fixing populations capable of replacing ammoniacal nitrogen and studying microbial ecology and soil diversity with successive enrichment steps of the same sample. Microbial biomass from N-fixer reactors at different concentrations allowed the tomato plants to grow and the chemical nitrogen-containing solution. Thus, packed bioreactors and the experimental framework, demonstrated for the first time in the current study, could play a future role in augmenting agricultural practices.

## Data Availability

The raw 16S rRNA gene amplicon sequencing data have been deposited in the European Nucleotide Archive and are available for download under project accession number PRJEB52882 (ERP137630). The associated sample accession number with this study is SAMEA14403571-SAMEA14403580. Additional data analyzed during this study are included in this published article (and its Supplementary Information Files).

## Supplementary Materials

The following supporting information can be downloaded at: https://www.mdpi.com/article/10.3390/microorganisms10071464/s1. Additionally File S1: Statistics for chemical parameters measured in bioreactors. Table S1. Summary of average physical and chemical parameters. Table S2. ANOVA coefficients for transformed OD600 response Table S3. ANOVA for biomass response coefficients for NT response. Statistics for chemical parameters measured in bioreactors. transformed biomass (mg/L). Table S4. ANOVA for COD % removal efficiency coefficients. Table S5. Coefficients for transformed Table S6. Comparing fertilizer treatment effects of the tomato growth rates in the first gathering Table S7. Tukey’s multiple pairwise comparisons of stem length in the first gathering. Table S8. Tukey’s multiple pairwise comparisons of stem length in the first gathering. Table S9. Tukey’s multiple pairwise comparisons of total length in the first gathering. Table S10. Tukey’s multiple pairwise comparisons of root width in the first gathering. Table S11. Tukey’s multiple pairwise comparisons of root volume in the first gathering. Table S12. Comparing fertilizer treatment effects of the tomato growth rates in the second gathering. Additional File S2: Phyloseq plots. Figure S1: Number of reads obtained after bioinformatic processing shown as (a) number of reads per OTU (or amplicon sequence variants—ASVs) and (b) the number of reads per sample. Figure S2: Rarefaction of final taxonomic data from microbiome analysis. Figure S3: Alpha diversity analysis using Observed, Chao1, ACE, Shannon, Simpson, and Inverse Simpson Metrics. Figure S4: Alpha diversity analysis using Observed, Chao1, ACE, Shannon, Simpson, and Inverse Simpson metrics for subset data. Figure S5: Heatmap showing the relative abundance of the top 20 phylum taxa identified from samples. Figure S6: Heatmap showing the relative abundance of the top 30 classes identified from samples. Figure S7: Heatmap showing the relative abundance of the top 40 order taxa identified from samples. Figure S8: Heatmap showing the relative abundance of the top 60 family taxa identified from samples. Figure S9: Heatmap showing the relative abundance of the top 80 genera identified from samples. Figure S10: Principal coordinate analysis (multidimensional scaling) of distance matrix using weighted UniFrac metrics. Coordinates for axes 1 and 2 are shown in the plot. Figure S11: Hierarchical clustering using UniFrac dissimilarity and UPGMA method. Figure S12: Hierarchical clustering using UniFrac dissimilarity and UPGMA method of Group A sample group in the present study. Figure S13: Hierarchical clustering using UniFrac dissimilarity and UPGMA method of Group B sample group in the present study. Figure S14: Phylogenetic tree using alignment and UPGMA method performed in muscle64. Figure S15: Phylogenetic tree using alignment and UPGMA method performed in muscle64 of two sample groups in the present study. Figure S16: Combined heat map and phylogenetic tree of ASVs with => 1000 total reads (*n* = 38) from samples. Figure S17: Combined heat map and phylogenetic tree of ASVs with => 500 total reads (*n* = 76) from samples. Additional File S3: Nitrogen cycle visualization. Table S1. Bioreactors main genus. Table S2. Soil main genus. Table S3. ASV-uniprot all matches. Table S4. Species UNIP. Table S5. Genus UNIP. Table S6. Enzymes and metabolism.
