# Supplementary material for: Exploiting the Potential of Bioreactors for Creating Spatial Organization in the Soil Microbiome: A Strategy for Increasing Sustainable Agricultural Practices
Source: Microorganisms. 2022 Jul 20;10(7):1464. doi: 10.3390/microorganisms10071464 (PMC9319577; doi:10.3390/microorganisms10071464)
Supplement: Supplementary file 1 [file microorganisms-10-01464-s001.zip › Additional file S1. Statistics figures.pdf]

Exploring soil microbiome self-assembling in bioreactors as a strategy to decrease chemical fertilizers in tomato plants

Carlos F. Gutiérrez[1], Nicolás Rodríguez-Romero[1], Siobhon Egan[2], Elaine Holmes[2], Janeth Sanabria[1,2*]

Statics for chemical parameters measured in Bioreactors

Table S1. Summary of average physical and chemical parameters.

		**Average values +/- Standard Deviation**	
		**Start a**	**Steady-state b**
OD600 (nm)	C-	0	0
	C+	0.28 +/- 0.15	0.59 +/- 0.09
	R2	0.39 +/- 0.24	0.76 +/- 0.1
	R3	0.41 +/- 0.23	0.78 +/- 0.09
	R5	0.28 +/- 0.15	0.59 +/- 0.09
Biomass (mg/L)	C-	0	0
	C+	0.09 +/- 0.04	541 +/- 457
	R2	0.1 +/- 0.04	1114 +/- 164
	R3	0.1 +/- 0.05	888 +/- 797
	R5	0.8 +/- 0.04	1223 +/- 1305
COD removal (%)	C-	18.57 +/- 9.84	13.01 +/- 2.06
	C+	57.92 +/- 30.97	95.87 +/- 0.64
	R2	56.92 +/- 32.01	97.98 +/- 0.36
	R3	61.56 +/- 30.43	84.01 +/- 0.16
	R5	59 +/- 29	88.28 +/- 4.38
TKN (mg/L)	C-	0.95 +/- 1.94	0.83
	C+	0.14 +/- 0.13	13.75
	R2	0.16 +/- 0.16	15
	R3	0.07 +/- 0.10	6.87
	R5	0.88 +/- 1.67	23.96

[a] Start refers to the process of reactors inoculation until they reach a steady-state after 80 days of operation. [b] Steady-state refers to the process in which there are no significant changes in the values and are relatively constant over time.

Table S1. Summary of average physical and chemical parameters.

		Average values +/- Standard Deviation	
		**Start a**	**Steady-state b**
N-NH4 (mg/L)	C-	1.14 +/- 1.87	0.2
	C+	0.53 +/- 2.16	0.08
	R2	1.55 +/- 2.35	0.1
	R3	1.67 +/- 2.48	0.07
	R5	1.97 +/- 2.56	0.08
N-NO3 (mg/L)	C-	0.06 +/- 0.11	0.01
	C+	0.06 +/- 0.1	0.43
	R2	0.07 +/- 0.1	0.14
	R3	0.07 +/- 0.1	0.09
	R5	0.08 +/- 0.11	0.84
N-NO2 (mg/L)	C-	0.02 +/- 0.01	0.09
	C+	0.02 +/- 0.01	0.11
	R2	0.02 +/- 0.01	0.12
	R3	0.02 +/- 0.02	0.14
	R5	0.02 +/- 0.01	0.14
N Fixed = TKN + NH4 + NO2 + NO3	C-	0.02 +/- 0.01	1.13
	C+	0.02 +/- 0.01	14.37
	R2	0.02 +/- 0.01	15.34
	R3	0.02 +/- 0.02	7.16
	R5	0.02 +/- 0.01	25.02

[a] Start refers to the process of reactors inoculation until they reach a steady-state after 80 days of operation. [b] Steady-state refers to the process in which there are no significant changes in the values and are relatively constant over time.

Table S2. Anova Coefficients for transformed OD_{600} response

Term	Coef	SE Coef.	T	p	VIF
**Constant**	-2,332	117	-19,99	0	
**Days**	1,683	197	8,54	0	2,00
**Treatment R**	-99	165	-0,60	548	3,42
		**Days * Treatment**			
**SAM**	0,00560	0,00279	2,01	0,047	4,42

**Equation of regression for OD_{600} response**	
**Treatments**	
**OD C+**	ln(Response) = -2,332 + 0,01683 Days
**OD SAM**	ln(Response) = -2,431 + 0,02243 Days

Table S3. ANOVA for biomass response coefficients for transformed biomass (mg/L)

Term	Coef	SE Coef.	T	p	VIF
**Constant**	-1,716	112	-15,30	0	
**Days_1**	767	159	4,81	0	2,00
**Treatment**					
**SAM**	8	159	0,05	960	2,14
**Days_1*Treatment**					
**SAM**	12	225	0,05	958	3,14
**Equation of regression for biomass response equation of regression**					
**Treatments**					
**C+**	-Response^-0,145643 = -1,716 + 0,00767 Days_1				
**SAM**	-Response^-0,145643 = -1,708 + 0,00779 Days_1				

Table S4. ANOVA for COD % removal efficiency coefficients

Term	Coef	SE Coef.	T	p	VIF
**Constant**	10,87	3,95	2,75	10	
**Days_1**	1,665	863	1,93	63	3,00
**Treatments**					
**C+**	6,46	5,59	1,16	257	4,76
**SAM**	2,02	5,59	0,36	720	4,76
**Days_1*Treatments**					
**C+**	868	122	7,11	0	5,43
**SAM**	889	122	7,29	0	5,43
**Equation of regression for COD % removal efficiency**					
**Treatments**					
**C+**	Response = 17,32 + 1,0343 Days				
**SAM**	Response = 12,89 + 1,0556 Days				

Table S5. Coefficients for transformed NT response

Term	Coef	SE Coef.	T	p	VIF
**Constant**	-1,53	1,01	-1,51	0,165	
**Days_1**	0,0014	0,0117	0,12	0,905	3,00
**Treatment**					
**C+**	-2,43	1,43	-1,70	0,123	2,67
**SAM**	-0,33	1,43	-0,23	0,823	2,67
**Days_1*Treatment**					
**C+**	0,0234	0,0166	1,41	0,193	3,33
**SAM**	0,0194	0,0166	1,17	0,272	3,33

Equation of regression for transformed NT

**Treatment**	
**C+**	ln(Response) = -3,96 + 0,0248 Days_1
**SAM**	ln(Response) = -1,85 + 0,0209 Days_1

Statics for chemical parameters measured in Bioreactors

Table S6. Comparing fertilizer treatment effects of the tomato growth rates in the first gathering.

Trend line equations and R square values
C- = 1,1117x - 0,3918 R^2 = 0,9982
C+ = 1,4638x - 0,8408 R^2 = 0,9802
P1 = 1,3189x + 0,3694 R^2 = 0,9709
P2 = 1,5791x - 0,398 R^2 = 0,9962
P3 = 1,2194x + 0,2286 R^2 = 0,9841

Table S7. Tukey multiple pairwise comparisons of Stem length in the first gathering.

		diff	lwr	upr	Padj
C+	-C	-2.7857143	-0.3451074	5.916536	0.0997065
P1	-C	-2.1	-1.0308216	5.230822	0.3163822
P2	-C	-3.5142857	0.3834641	6.645107	**0.0217685**
P3	-C	-1.7285714	-1.4022502	4.859393	0.5080016
P1	-C+	-0.6857143	-3.8165359	2.445107	0.9680413
P2	-C	+0.7285714	-2.4022502	3.859393	0.9603222
P3	-C+	-1.0571429	-4.1879645	2.073679	0.8623589
P2	-P1	1.4142857	-1.7165359	4.545107	0.6870557
P3	-P1	-0.3714286	-3.5022502	2.759393	0.9968156
P3	-P2	-1.7857143	-4.9165359	1.345107	0.4760769

Table S8. Tukey multiple pairwise comparisons of Stem length in the first gathering.

	diff	lwr	upr	padj
**C+-C-**	1.3714286	-0.4462773	3.18913445	0.2115377
**P1-C-**	2.0000000	0.1822941	3.81770588	**0.0254190**
**P2-C-**	2.5285714	0.7108655	4.34627731	**0.0029723**
**P3-C-**	0.7000000	-1.1177059	2.51770588	0.7963371
**P1-C+**	0.6285714	-1.1891345	2.44627731	0.8518911
**P2-C+**	1.1571429	-0.660563	2.97484874	0.367049
**P3-C+**	-0.6714286	-2.4891345	1.14627731	0.8195156
**P2-P1**	0.5285714	-1.2891345	2.34627731	0.9147368
**P3-P1**	-1.3000000	-3.1177059	0.51770588	0.2572031
**P3-P2**	-1.8285714	-3.6462773	-0.01086555	**0.0480815**

Table S9. Tukey multiple pairwise comparisons of Total length in the first gathering.

	diff	lwr	upr	padj
**C+-C-**	3.600000	-0.9800697	8.180070	0.1794070
**P1-C-**	4.000001	-0.4800697	8.680070	0.0965278
**P2-C-**	6.042857	1.4627875	10.622927	**0.0051454**
**P3-C-**	2.428571	-2.1514983	7.008641	0.5468984
**P1-C+**	0.500000	-4.0800697	5.080070	0.9976950
**P2-C+**	2.442857	-2.1372125	7.022927	0.5413182
**P3-C+**	-1.171429	-5.7514983	3.408641	0.9447722
**P2-P1**	1.942857	-2.6372125	6.522927	0.7340233
**P3-P1**	-1.671429	-6.2514983	2.908641	0.8258500
**P3-P2**	-3.614286	-8.1943554	0.965784	0.1764419

Table S10. Tukey multiple pairwise comparisons of Root width in the first gathering.

	diff	lwr	upr	P adj
**C+-C-**	0.32857143	-0.8764859	1.53362876	0.9312654
**P1-C-**	0.78571429	-0.41934304	1.99077161	0.3436463
**P2-C-**	1.62857143	0.4235141	2.83362876	**0.0040306**
**P3-C-**	0.38571429	-0.81934304	1.59077161	0.8835997
**P1-C+**	0.45714286	-0.74791447	1.66220019	0.8049507
**P2-C+**	1.30000000	0.09494267	2.50505733	**0.0294874**
**P3-C+**	0.05714286	-1.14791447	1.26220019	0.9999144
**P2-P1**	0.84285714	-0.36220019	2.04791447	0.2772783
**P3-P1**	-0.4000000	-1.60505733	0.80505733	0.8694778
**P3-P2**	-1.24285714	-2.44791447	-0.03779981	**0.0406474**

Table S11. Tukey multiple pairwise comparisons of Root volume in the first gathering.

	diff	lwr	upr	padj
**C+-C-**	2.4549360	-10.64491224	15.554784	0.9819044
**P1-C-**	6.6637450	-6.43610324	19.763593	0.5856284
**P2-C-**	15.6326016	2.53275336	28.732450	**0.0131211**
**P3-C-**	2.9239694	-10.17587884	16.023818	0.9657966
**P1-C+**	4.2088090	-8.89103924	17.308657	0.8822008
**P2-C+**	13.1776656	0.07781736	26.277514	**0.0480932**
**P3-C+**	0.4690334	-12.63081484	13.568882	0.9999720
**P2-P1**	8.9688566	-4.13099164	22.068705	0.2970215
**P3-P1**	-3.7397756	-16.83962384	9.360073	0.9197947
**P3-P2**	-12.7086322	-25.80848044	0.391216	**0.0606439**

Table S12. Comparing fertilizer treatment effects of the tomato growth rates in the second gathering.

**C+** = -0,002x4 + 0,0718x3 - 0,3251x2 + 0,2514x + 3,6092 R^2 = 0,9978
**P1** = -0,0021x4 + 0,093x3 - 0,8747x2 + 3,8588x - 2,0546 R^2 = 0,9992
**P2** = -0,0049x4 + 0,2217x3 - 2,7156x2 + 12,122x - 12,06 R^2 = 0,9976
**P3** = -0,0007x4 + 0,0227x3 + 0,2141x2 - 1,9876x + 4,8939 R^2 = 0,9955
