# Supplementary material for: Exploiting the Potential of Bioreactors for Creating Spatial Organization in the Soil Microbiome: A Strategy for Increasing Sustainable Agricultural Practices
Source: Microorganisms. 2022 Jul 20;10(7):1464. doi: 10.3390/microorganisms10071464 (PMC9319577; doi:10.3390/microorganisms10071464)
Supplement: Supplementary file 1 [file microorganisms-10-01464-s001.zip › Additional file S2. phyloseq plots.pdf]

# Exploiting the potential of bioreactors for creating spatial organisation in the soil microbiome: A strategy for increasing sustainable agricultural practices

**Authors:** Carlos F. Gutiérrez[1], Nicolás Rodríguez-Romero[1], Siobhon Egan[2], Elaine Holmes[2] and Janeth Sanabria[1,2] *

1. Environmental Microbiology and Biotechnology Laboratory, Engineering School of Environmental & Natural Resources, Engineering Faculty, Universidad del Valle – Meléndez campus, Cali, Colombia.
2. Australian National Phenome Center, Murdoch University, Perth Australia

- Correspondence: janeth.sanabria@correounivalle.edu.co

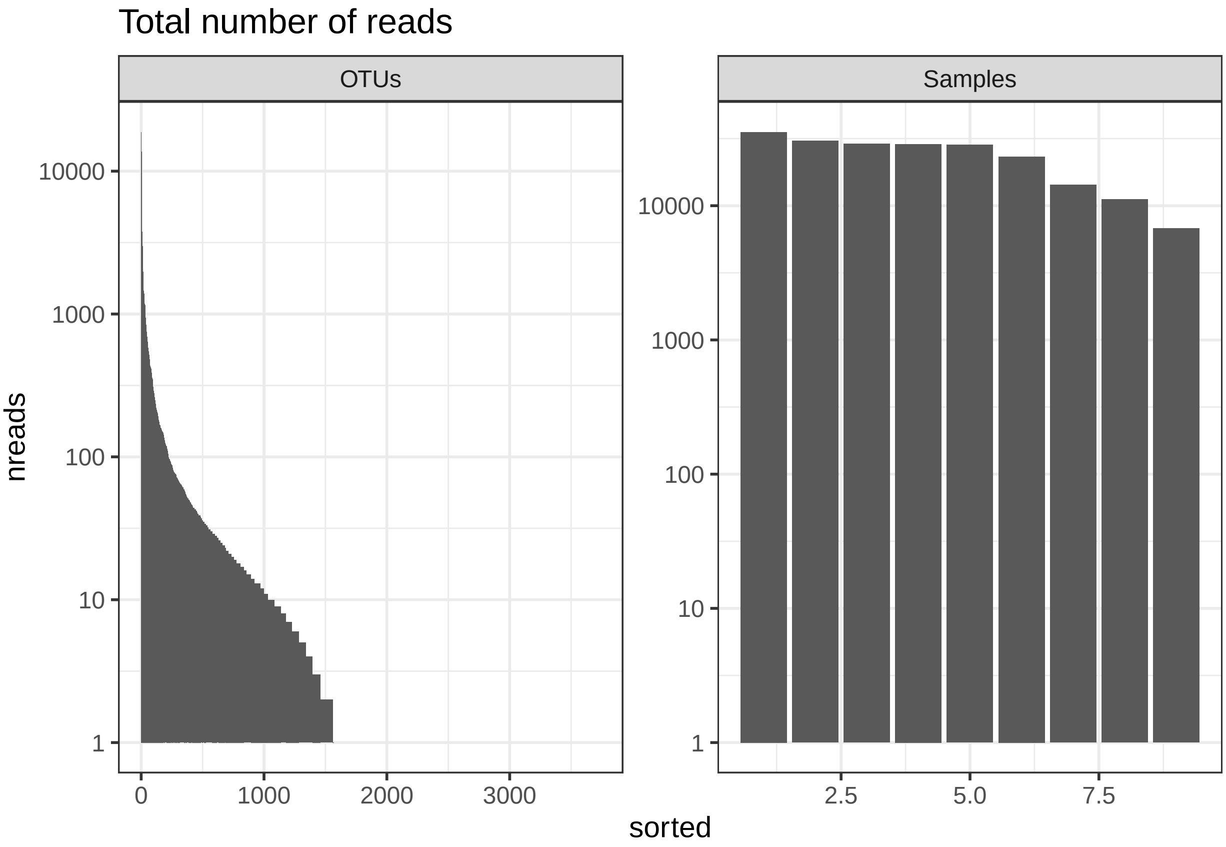


Figure S1: Number of reads obtained after bioinformatic processing shown as (a) number of reads per OTU (or amplicon sequence variants - ASVs) and (b) number of reads per sample.

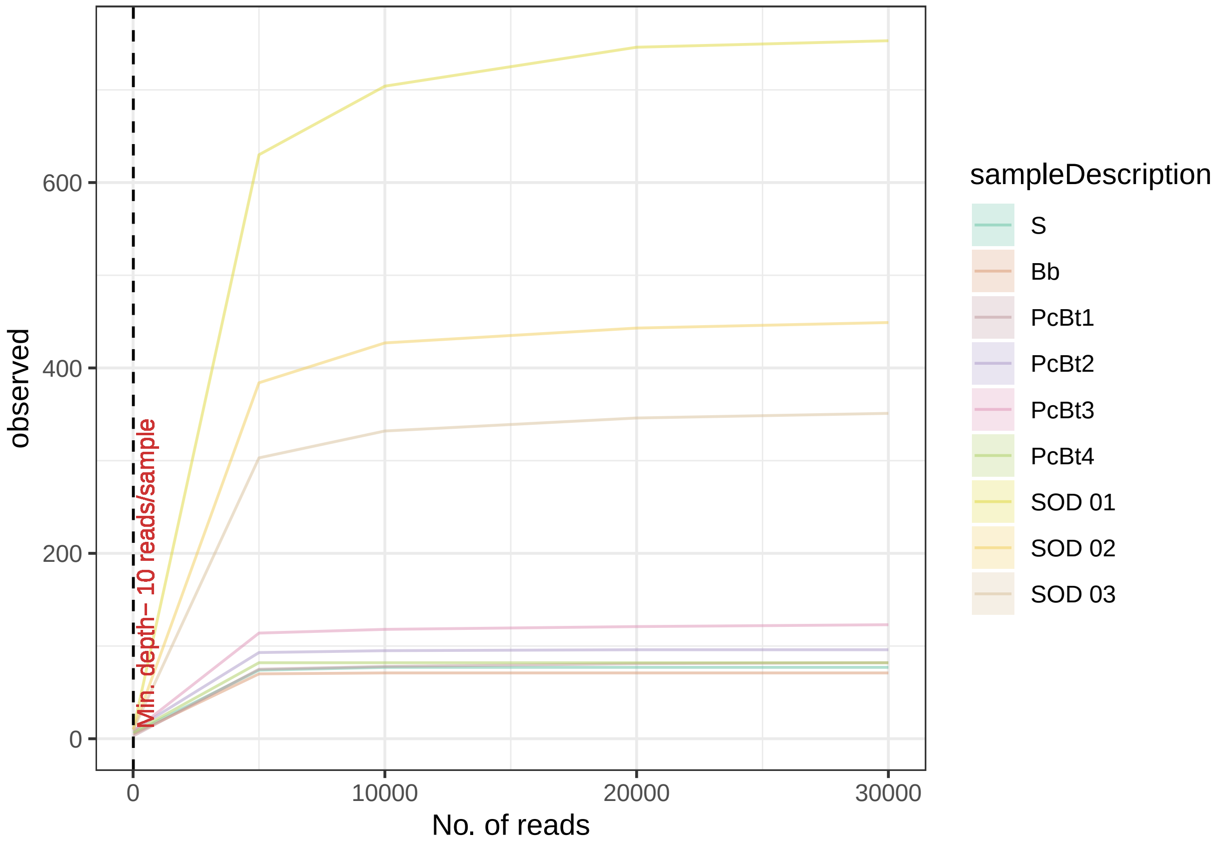


Figure S2: Rarefaction of final taxonomic data from microbiome analysis

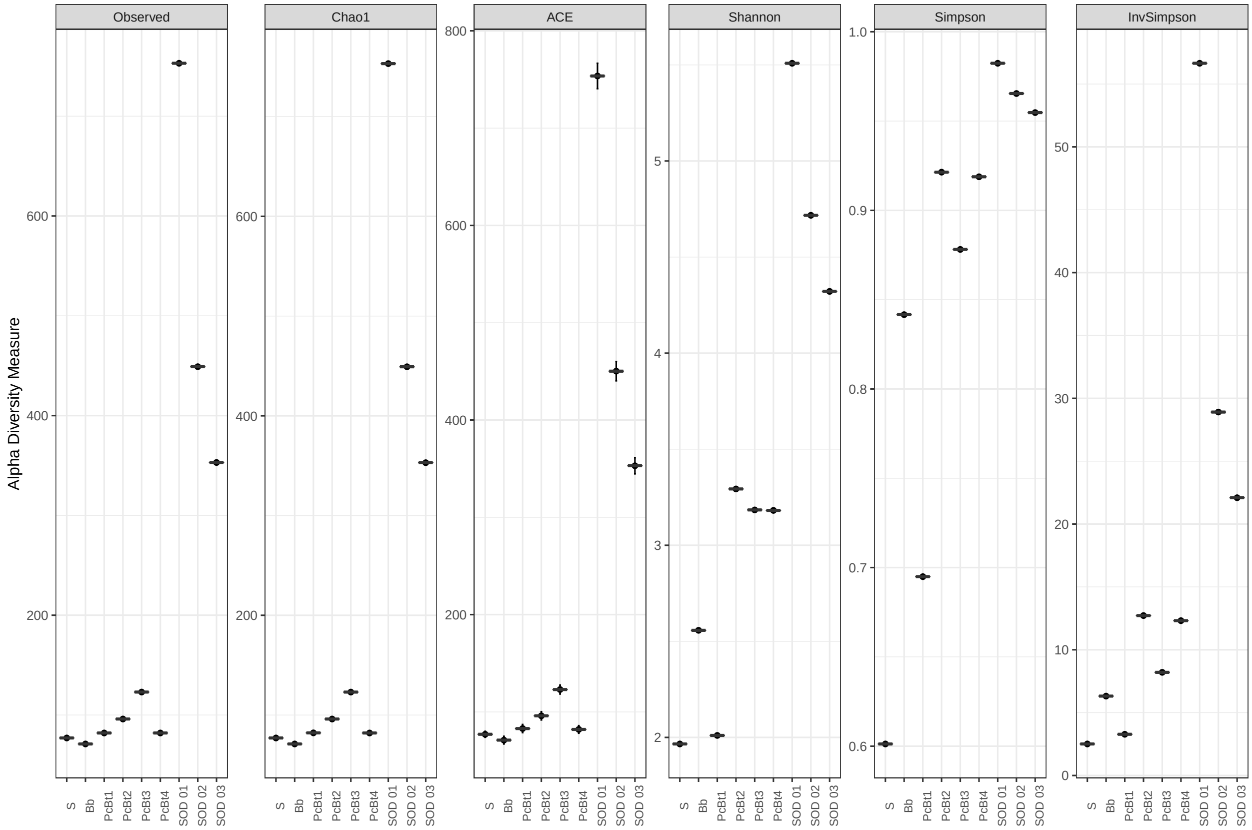


Figure S3: Alpha diveristy analysis using Observed, Chao1, ACE, Shannon, Simpson and Inverse Simpson metrics

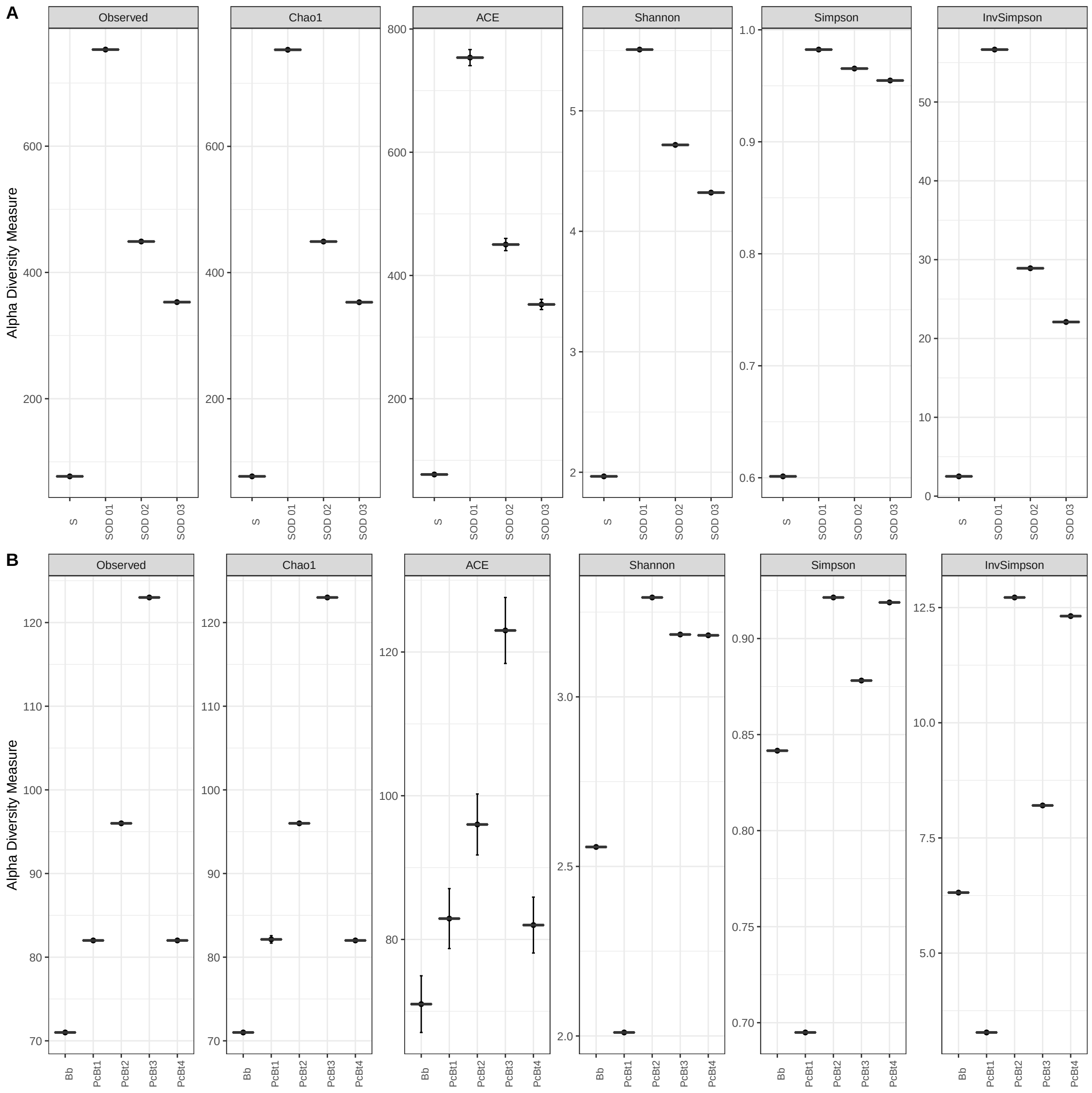


Figure S4: Alpha diveristy analysis using Observed, Chao1, ACE, Shannon, Simpson and Inverse Simpson metrics for subset data

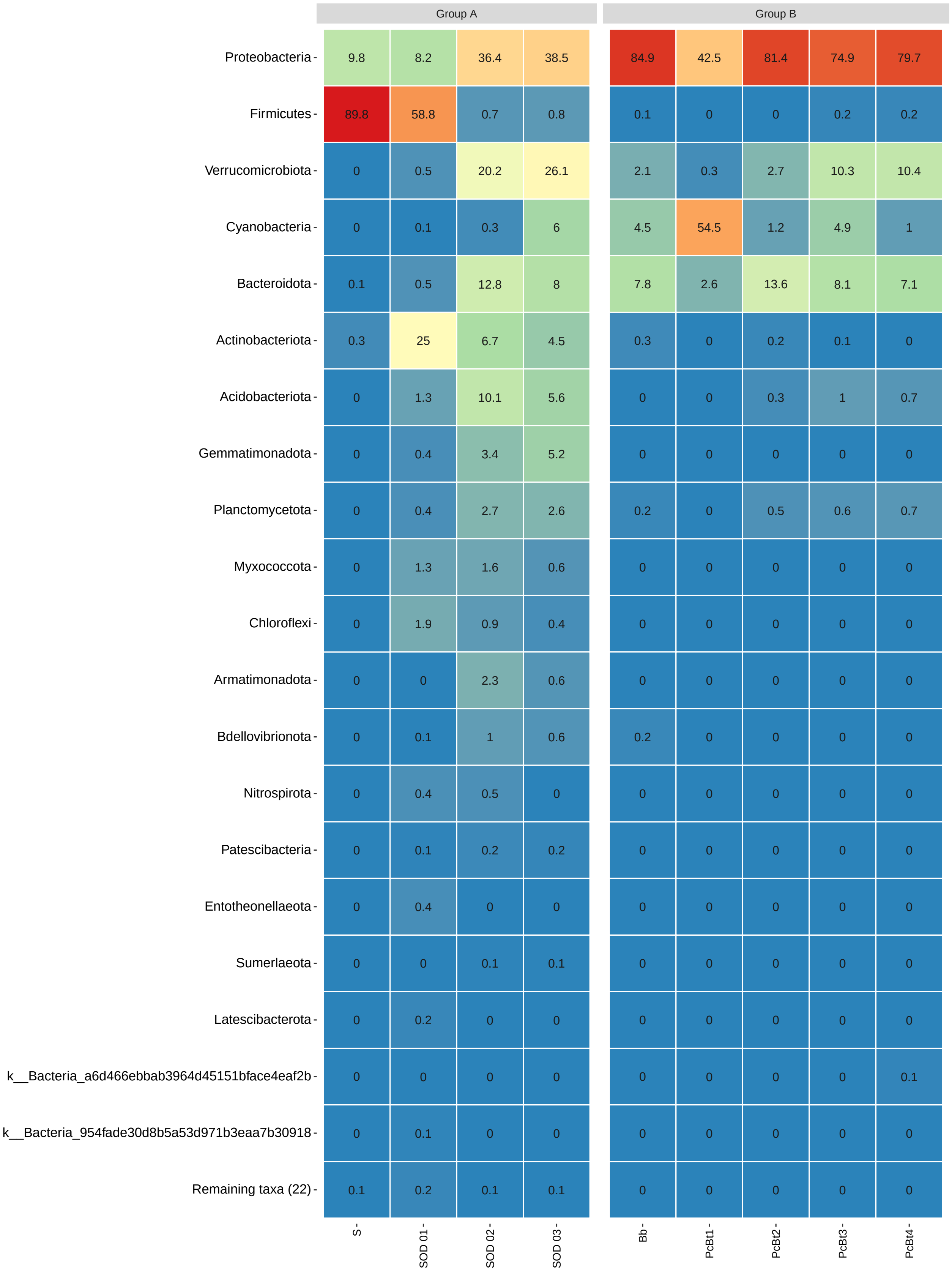


Figure S5: Heatmap showing relative abundance of top 20 phylum taxa identified from samples

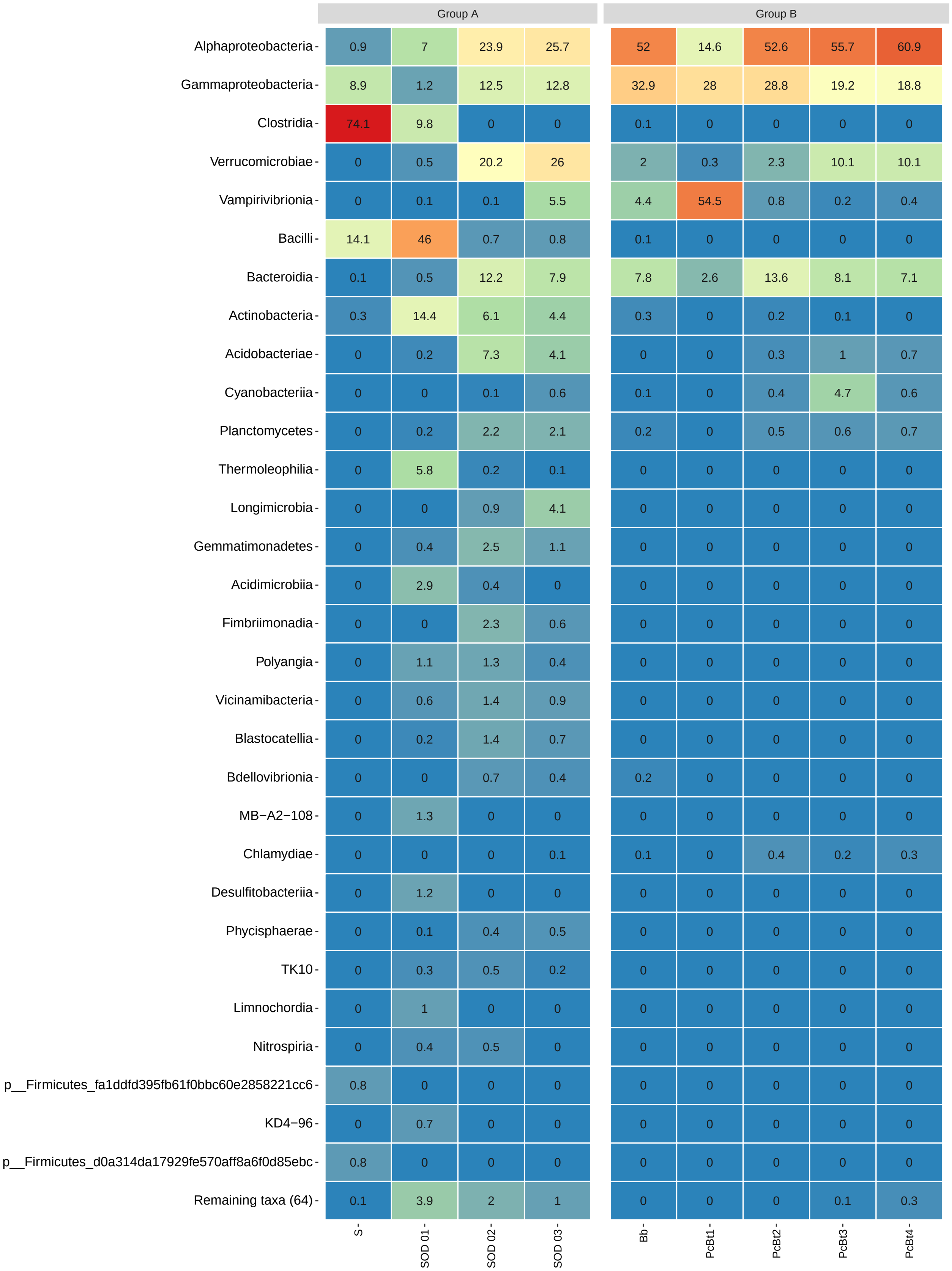


Figure S6: Heatmap showing relative abundance of top 30 class identified from samples

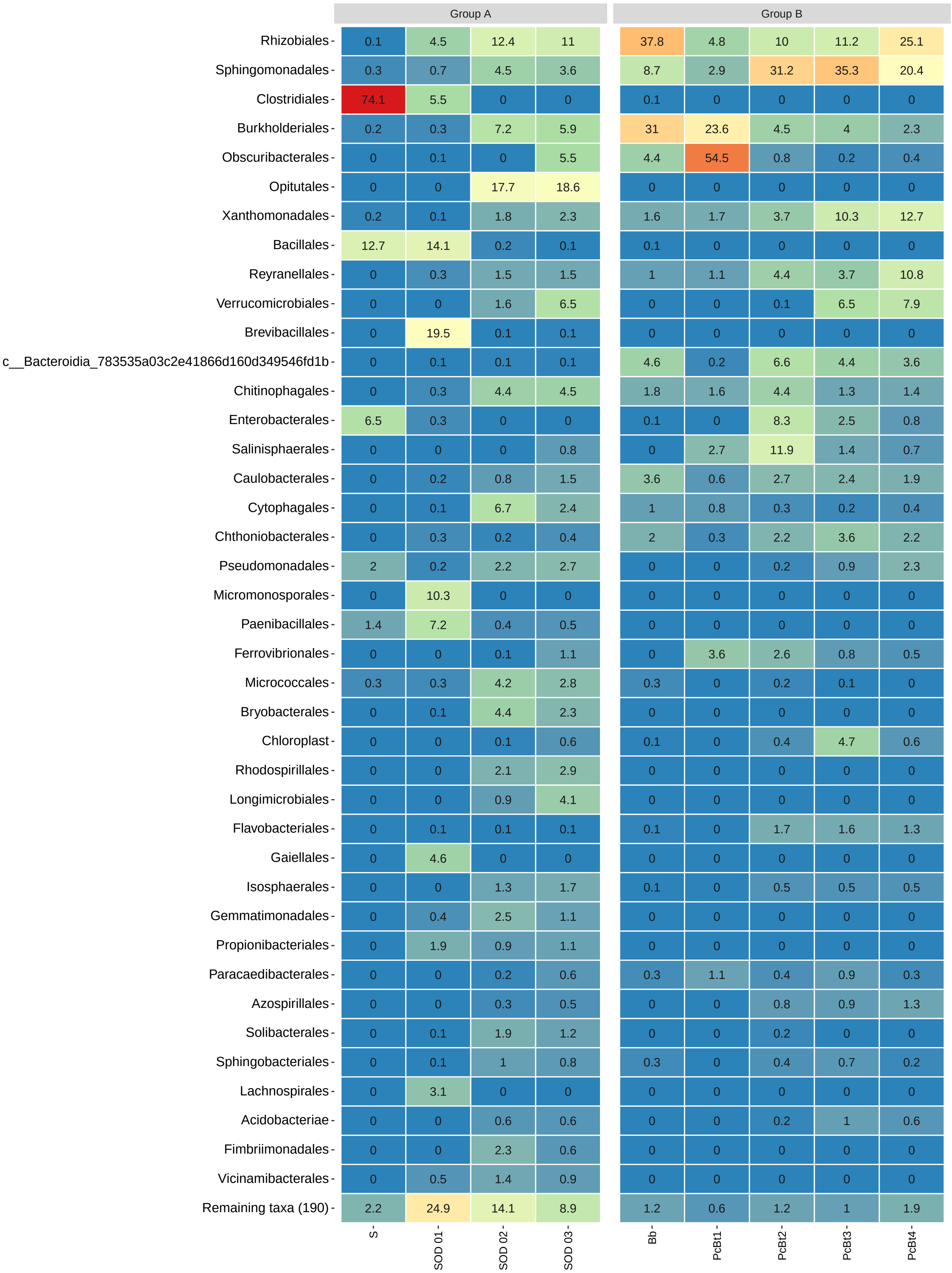


Figure S7: Heatmap showing relative abundance of top 40 order taxa identified from samples

| | Group A | | | | Group B | | | | |
|---|---|---|---|---|---|---|---|---|---|
| | S | SOD 01 | SOD 02 | SOD 03 | Bb | PcBt1 | PcBt2 | PcBt3 | PcBt4 |
| Sphingomonadaceae | 0.3 | 0.7 | 4.5 | 3.6 | 8.7 | 2.9 | 31.2 | 35.3 | 20.4 |
| Clostridiaceae | 74.1 | 4.5 | 0 | 0 | 0.1 | 0 | 0 | 0 | 0 |
| Obscuribacteraceae | 0 | 0.1 | 0 | 5.5 | 4.4 | 54.5 | 0.8 | 0.2 | 0.4 |
| Rhodocyclaceae | 0 | 0 | 0.6 | 2.5 | 18.7 | 22.8 | 2.3 | 1.9 | 1.2 |
| Beijerinckiaceae | 0 | 0.5 | 2.4 | 1.8 | 34.2 | 2.5 | 2.2 | 1.4 | 0.8 |
| Xanthobacteraceae | 0 | 2.9 | 3.7 | 4 | 1 | 0.7 | 4.9 | 5.9 | 17.9 |
| Opitutaceae | 0 | 0 | 17.7 | 18.6 | 0 | 0 | 0 | 0 | 0 |
| Xanthomonadaceae | 0.2 | 0.1 | 1.7 | 2.2 | 1.6 | 1.7 | 3.7 | 10.3 | 12.7 |
| Reyranellaceae | 0 | 0.3 | 1.5 | 1.5 | 1 | 1.1 | 4.4 | 3.7 | 10.8 |
| Comamonadaceae | 0 | 0.1 | 4.7 | 1.8 | 11.1 | 0.7 | 2.1 | 2 | 1.1 |
| Verrucomicrobiaceae | 0 | 0 | 1.5 | 6.5 | 0 | 0 | 0.1 | 6.5 | 7.9 |
| Brevibacillaceae | 0 | 19.5 | 0.1 | 0.1 | 0 | 0 | 0 | 0 | 0 |
| c__Bacteroidia_783535a03c2e41866d160d349546fd1b | 0 | 0.1 | 0.1 | 0.1 | 4.6 | 0.2 | 6.6 | 4.4 | 3.6 |
| Chitinophagaceae | 0 | 0.2 | 4.3 | 4.5 | 1.8 | 1.6 | 4.4 | 1.3 | 1.4 |
| Enterobacteriaceae | 6.3 | 0.3 | 0 | 0 | 0.1 | 0 | 8.3 | 2.5 | 0.8 |
| Solimonadaceae | 0 | 0 | 0 | 0.8 | 0 | 2.7 | 11.9 | 1.4 | 0.7 |
| Bacillaceae | 0.8 | 13.2 | 0.2 | 0.1 | 0 | 0 | 0 | 0 | 0 |
| Rhizobiaceae | 0.1 | 0.4 | 2.7 | 2.2 | 1.3 | 0.2 | 1.3 | 2.7 | 3.4 |
| Sporolactobacillaceae | 11.6 | 0.9 | 0 | 0 | 0.1 | 0 | 0 | 0 | 0 |
| Terrimicrobiaceae | 0 | 0 | 0.1 | 0.4 | 2 | 0.3 | 2.2 | 3.6 | 2.2 |
| Pseudomonadaceae | 2 | 0.2 | 2.2 | 2.7 | 0 | 0 | 0.2 | 0.9 | 2.3 |
| Caulobacteraceae | 0 | 0.1 | 0.5 | 0.5 | 3.5 | 0.6 | 2.5 | 1.7 | 0.9 |
| Micromonosporaceae | 0 | 10.3 | 0 | 0 | 0 | 0 | 0 | 0 | 0 |
| Paenibacillaceae | 1.4 | 7.2 | 0.4 | 0.5 | 0 | 0 | 0 | 0 | 0 |
| Ferrovibrionaceae | 0 | 0 | 0.1 | 1.1 | 0 | 3.6 | 2.6 | 0.8 | 0.5 |
| Microscillaceae | 0 | 0.1 | 6.4 | 1.9 | 0 | 0 | 0 | 0 | 0 |
| Bryobacteraceae | 0 | 0.1 | 4.4 | 2.3 | 0 | 0 | 0 | 0 | 0 |
| Chloroplast | 0 | 0 | 0.1 | 0.6 | 0.1 | 0 | 0.4 | 4.7 | 0.6 |
| Intrasporangiaceae | 0 | 0.1 | 2.8 | 2.5 | 0 | 0 | 0 | 0 | 0 |
| Rhizobiales_Incertae_Sedis | 0 | 0.1 | 1.2 | 0.7 | 0.3 | 1.1 | 0.3 | 0.2 | 1.3 |
| Longimicrobiaceae | 0 | 0 | 0.9 | 4.1 | 0 | 0 | 0 | 0 | 0 |
| Hyphomicrobiaceae | 0 | 0.3 | 1.2 | 1.5 | 0.2 | 0.1 | 0.5 | 0.4 | 0.5 |
| Isosphaeraceae | 0 | 0 | 1.3 | 1.7 | 0.1 | 0 | 0.5 | 0.5 | 0.5 |
| Weeksellaceae | 0 | 0 | 0 | 0 | 0.1 | 0 | 1.4 | 1.5 | 1.3 |
| o__Rhodospirillales_e40fa32b862876e8d638ff2c4d3ed663 | 0 | 0 | 1.7 | 2.6 | 0 | 0 | 0 | 0 | 0 |
| Gemmatimonadaceae | 0 | 0.4 | 2.5 | 1.1 | 0 | 0 | 0 | 0 | 0 |
| Paracaedibacteraceae | 0 | 0 | 0.2 | 0.6 | 0.3 | 1.1 | 0.4 | 0.9 | 0.3 |
| Azospirillaceae | 0 | 0 | 0.2 | 0.5 | 0 | 0 | 0.8 | 0.9 | 1.3 |
| Nocardioidaceae | 0 | 1.5 | 0.9 | 1.1 | 0 | 0 | 0 | 0 | 0 |
| Solibacteraceae | 0 | 0.1 | 1.9 | 1.2 | 0 | 0 | 0.2 | 0 | 0 |
| Methylopilaceae | 0 | 0 | 0 | 0.1 | 0.7 | 0.3 | 0.8 | 0.7 | 0.8 |
| Hyphomonadaceae | 0 | 0 | 0.3 | 1 | 0.1 | 0 | 0.2 | 0.7 | 1 |
| Lachnospiraceae | 0 | 3.1 | 0 | 0 | 0 | 0 | 0 | 0 | 0 |
| Spirosomaceae | 0 | 0 | 0.1 | 0.4 | 1 | 0.8 | 0.3 | 0.2 | 0.4 |
| Acidobacteriae | 0 | 0 | 0.6 | 0.6 | 0 | 0 | 0.2 | 1 | 0.6 |
| Fimbriimonadaceae | 0 | 0 | 2.3 | 0.6 | 0 | 0 | 0 | 0 | 0 |
| Thermoactinomycetaceae | 0 | 2.6 | 0 | 0 | 0 | 0 | 0 | 0 | 0 |
| Caedibacteraceae | 0 | 0 | 0.1 | 0.4 | 0.4 | 0.4 | 0.2 | 0.3 | 0.5 |
| Burkholderiaceae | 0.2 | 0 | 0.9 | 1.2 | 0 | 0 | 0 | 0 | 0 |
| Acetobacteraceae | 0.5 | 0.1 | 0.4 | 0.7 | 0.1 | 0 | 0.1 | 0.1 | 0.2 |
| Devosiaceae | 0 | 0 | 0.9 | 0.6 | 0.2 | 0 | 0 | 0 | 0.4 |
| Blastocatellaceae | 0 | 0 | 1.4 | 0.7 | 0 | 0 | 0 | 0 | 0 |
| Vicinamibacteraceae | 0 | 0.4 | 1 | 0.6 | 0 | 0 | 0 | 0 | 0 |
| Sphingobacteriaceae | 0 | 0 | 0.4 | 0.1 | 0.3 | 0 | 0.4 | 0.7 | 0.2 |
| Micrococcaceae | 0 | 0.1 | 1.2 | 0.3 | 0 | 0 | 0 | 0 | 0 |
| Streptomycetaceae | 0 | 0.9 | 0.6 | 0 | 0 | 0 | 0 | 0 | 0 |
| Pedosphaeraceae | 0 | 0.2 | 0.8 | 0.4 | 0 | 0 | 0 | 0 | 0 |
| Rhodobacteraceae | 0 | 0 | 0.4 | 1 | 0 | 0 | 0 | 0 | 0 |
| Methylophilaceae | 0 | 0 | 0 | 0 | 1.2 | 0.1 | 0 | 0.1 | 0 |
| MB−A2−108 | 0 | 1.3 | 0 | 0 | 0 | 0 | 0 | 0 | 0 |
| Remaining taxa (345) | 2.5 | 27.1 | 13.7 | 7.7 | 1 | 0.2 | 1.4 | 0.9 | 1.3 |

Figure S8: Heatmap showing relative abundance of top 60 family taxa identified from samples

| | Group A | | | | Group B | | | | |
|---|---|---|---|---|---|---|---|---|---|
| | S | SOD 01 | SOD 02 | SOD 03 | Bb | PcBt1 | PcBt2 | PcBt3 | PcBt4 |
| Sphingobium | 0.1 | 0.1 | 0.2 | 0.2 | 0.7 | 0.8 | 21.7 | 32.6 | 17.5 |
| Clostridium_sensu_stricto_12 | 68.2 | 3 | 0 | 0 | 0.1 | 0 | 0 | 0 | 0 |
| Candidatus_Obscuribacter | 0 | 0.1 | 0 | 5.5 | 4.4 | 54.3 | 0.7 | 0.1 | 0.1 |
| Zoogloea | 0 | 0 | 0.6 | 2.5 | 18.1 | 22.8 | 0 | 0 | 0 |
| Beijerinckia | 0 | 0.2 | 0.1 | 0.9 | 33.4 | 2.5 | 2 | 1.3 | 0.8 |
| Opitutus | 0 | 0 | 17.2 | 18.3 | 0 | 0 | 0 | 0 | 0 |
| Pseudoxanthomonas | 0 | 0 | 1.3 | 2 | 1.5 | 1.7 | 3.7 | 10.1 | 12.2 |
| Xanthobacter | 0 | 0 | 1.3 | 2.4 | 0.5 | 0.3 | 4.2 | 5.2 | 17.9 |
| Reyranella | 0 | 0.3 | 1.4 | 1.4 | 1 | 1.1 | 4.4 | 3.7 | 10.8 |
| Brevibacillus | 0 | 19.5 | 0.1 | 0.1 | 0 | 0 | 0 | 0 | 0 |
| c__Bacteroidia_783535a03c2e41866d160d349546fd1b | 0 | 0.1 | 0.1 | 0.1 | 4.6 | 0.2 | 6.6 | 4.4 | 3.6 |
| Hydrocarboniphaga | 0 | 0 | 0 | 0.7 | 0 | 2.7 | 11.9 | 1.4 | 0.7 |
| Novosphingobium | 0.1 | 0.1 | 0 | 0.3 | 7.4 | 1.1 | 6.7 | 0.7 | 0.4 |
| Prosthecobacter | 0 | 0 | 0.2 | 0.1 | 0 | 0 | 0.1 | 6.5 | 7.9 |
| Bacillus | 0.8 | 12.3 | 0.2 | 0.1 | 0 | 0 | 0 | 0 | 0 |
| Sporolactobacillus | 11.5 | 0.9 | 0 | 0 | 0.1 | 0 | 0 | 0 | 0 |
| Sphingomonas | 0 | 0.3 | 3.3 | 2.1 | 0 | 0.5 | 2.4 | 1.5 | 1.5 |
| Terrimicrobium | 0 | 0 | 0.1 | 0.4 | 2 | 0.3 | 2.2 | 3.6 | 2.2 |
| Pelomonas | 0 | 0 | 0 | 0 | 10.1 | 0.1 | 0 | 0 | 0 |
| Ferrovibrio | 0 | 0 | 0.1 | 1.1 | 0 | 3.6 | 2.6 | 0.8 | 0.5 |
| f__Chitinophagaceae_9d9e4ad8ee3d7e910f6237bb14bcc4da | 0 | 0 | 0.5 | 1 | 0 | 0.3 | 3.9 | 1 | 1.4 |
| Pseudomonas | 0 | 0 | 2.2 | 2.4 | 0 | 0 | 0.2 | 0.9 | 2.3 |
| Roseimicrobium | 0 | 0 | 1.3 | 6.2 | 0 | 0 | 0 | 0 | 0 |
| Bryobacter | 0 | 0.1 | 4.4 | 2.3 | 0 | 0 | 0 | 0 | 0 |
| Clostridium_sensu_stricto_1 | 5.5 | 1.2 | 0 | 0 | 0 | 0 | 0 | 0 | 0 |
| Chloroplast | 0 | 0 | 0.1 | 0.6 | 0.1 | 0 | 0.4 | 4.7 | 0.6 |
| f__Microscillaceae_d4802ec1ee4dc6981e399d101ce8a8d5 | 0 | 0 | 5.3 | 1.2 | 0 | 0 | 0 | 0 | 0 |
| f__Enterobacteriaceae_527a09644f077fbcef30effba12056ff | 0 | 0 | 0 | 0 | 0 | 0 | 3.4 | 1.1 | 0.8 |
| Paenibacillus | 1.4 | 2.8 | 0.3 | 0.5 | 0 | 0 | 0 | 0 | 0 |
| Longimicrobium | 0 | 0 | 0.9 | 4.1 | 0 | 0 | 0 | 0 | 0 |
| Nordella | 0 | 0.1 | 1 | 0.6 | 0.3 | 1.1 | 0.3 | 0.2 | 1.3 |
| Terrimonas | 0 | 0 | 0.8 | 0.9 | 1.8 | 1 | 0 | 0.1 | 0 |
| Cloacibacterium | 0 | 0 | 0 | 0 | 0.1 | 0 | 1.4 | 1.5 | 1.3 |
| o__Rhodospirillales_e40fa32b862876e8d638ff2c4d3ed663 | 0 | 0 | 1.7 | 2.6 | 0 | 0 | 0 | 0 | 0 |
| Phenylobacterium | 0 | 0.1 | 0.2 | 0.3 | 3 | 0.1 | 0.1 | 0.1 | 0.2 |
| Azonexus | 0 | 0 | 0 | 0 | 0.4 | 0 | 1.8 | 1.1 | 0.6 |
| f__Isosphaeraceae_136d9eb41806e28a7dfc193424250524 | 0 | 0 | 1.1 | 1.5 | 0.1 | 0 | 0.5 | 0.4 | 0.3 |
| Gemmatimonas | 0 | 0.2 | 2.3 | 1.1 | 0 | 0 | 0 | 0 | 0 |
| Brevundimonas | 0 | 0 | 0.2 | 0 | 0.3 | 0 | 1.1 | 1.1 | 0.8 |
| f__Intrasporangiaceae_0cf152dc807b570c5f5ab1a7aea69901 | 0 | 0 | 1.3 | 2.2 | 0 | 0 | 0 | 0 | 0 |
| Candidatus_Finniella | 0 | 0 | 0 | 0.4 | 0.3 | 1.1 | 0.4 | 0.9 | 0.3 |
| Sphingopyxis | 0 | 0 | 0.2 | 0.3 | 0.6 | 0.5 | 0.4 | 0.5 | 1 |
| Candidatus_Solibacter | 0 | 0.1 | 1.9 | 1.2 | 0 | 0 | 0.2 | 0 | 0 |
| Methylopila | 0 | 0 | 0 | 0.1 | 0.7 | 0.3 | 0.8 | 0.7 | 0.8 |
| Aminobacter | 0 | 0 | 0.2 | 0.1 | 0.6 | 0.1 | 0.4 | 0.8 | 1 |
| f__Enterobacteriaceae_e8b77cf2f7dd1f17a7c7626f2904d7b3 | 2.9 | 0.3 | 0 | 0 | 0 | 0 | 0 | 0 | 0 |
| Hyphomonas | 0 | 0 | 0.3 | 0.9 | 0 | 0 | 0.2 | 0.7 | 1 |
| f__Rhizobiaceae_90a634fef584638a5861ac4f26074d88 | 0 | 0 | 0.2 | 0.2 | 0 | 0 | 0.6 | 0.9 | 1.1 |
| Paludibaculum | 0 | 0 | 0.6 | 0.6 | 0 | 0 | 0.2 | 1 | 0.6 |
| Fimbriimonadaceae | 0 | 0 | 2.3 | 0.6 | 0 | 0 | 0 | 0 | 0 |
| Micromonospora | 0 | 2.9 | 0 | 0 | 0 | 0 | 0 | 0 | 0 |
| Fibrisoma | 0 | 0 | 0 | 0.2 | 1 | 0.8 | 0.3 | 0.2 | 0.4 |
| Anaerocolumna | 0 | 2.7 | 0 | 0 | 0 | 0 | 0 | 0 | 0 |
| f__Enterobacteriaceae_97822797c63078d0e598100e24f71b7d | 0 | 0 | 0 | 0 | 0 | 0 | 1.7 | 1.1 | 0 |
| Caulobacter | 0 | 0 | 0.1 | 0.2 | 0.2 | 0.5 | 1.2 | 0.4 | 0 |
| Pedomicrobium | 0 | 0.3 | 1.1 | 1.2 | 0 | 0 | 0 | 0 | 0 |
| Klebsiella | 2.4 | 0 | 0 | 0 | 0 | 0 | 0 | 0 | 0 |
| Pseudacidovorax | 0 | 0 | 0 | 0 | 0 | 0 | 1 | 0.9 | 0.6 |
| Hyphomicrobium | 0 | 0 | 0.2 | 0.3 | 0.2 | 0.1 | 0.5 | 0.4 | 0.5 |
| f__Comamonadaceae_61f7050e1641b84a11835f862bb20726 | 0 | 0 | 0 | 0.3 | 0.9 | 0.5 | 0.3 | 0.1 | 0.2 |
| Candidatus_Nucleicultrix | 0 | 0 | 0.1 | 0.4 | 0.4 | 0.4 | 0.2 | 0.3 | 0.5 |
| Azospirillum | 0 | 0 | 0.2 | 0.4 | 0 | 0 | 0 | 0.5 | 1 |
| Flavitalea | 0 | 0 | 1.3 | 0.9 | 0 | 0 | 0 | 0 | 0 |
| f__Enterobacteriaceae_15318ef23ab68840e3368e4d318f95ad | 0 | 0 | 0 | 0 | 0.1 | 0 | 1.7 | 0.3 | 0 |
| f__Xanthobacteraceae_919ef4fa379e506a07d1d74ac3b7faba | 0 | 0 | 0 | 0.2 | 0.3 | 0.3 | 0.6 | 0.6 | 0 |
| Azospira | 0 | 0 | 0 | 0 | 0.1 | 0 | 0.4 | 0.8 | 0.6 |
| Cupriavidus | 0 | 0 | 0.9 | 1.1 | 0 | 0 | 0 | 0 | 0 |
| Comamonas | 0 | 0 | 0 | 0 | 0 | 0 | 0.8 | 0.9 | 0.2 |
| Flavihumibacter | 0 | 0 | 0.6 | 1.3 | 0 | 0 | 0 | 0 | 0 |
| f__Comamonadaceae_15157002701d572334bc12450e742d75 | 0 | 0 | 1.8 | 0 | 0 | 0 | 0 | 0 | 0 |
| Shinella | 0 | 0.1 | 0.6 | 0.2 | 0 | 0 | 0 | 0.4 | 0.5 |
| Devosia | 0 | 0 | 0.6 | 0.6 | 0.2 | 0 | 0 | 0 | 0.4 |
| Vicinamibacteraceae | 0 | 0.4 | 0.8 | 0.5 | 0 | 0 | 0 | 0 | 0 |
| Hydrogenophaga | 0 | 0 | 0.6 | 1.1 | 0 | 0 | 0 | 0 | 0 |
| f__Pseudomonadaceae_5953c5b70f425e1d94a03e92aebcb6c7 | 1.5 | 0.2 | 0 | 0 | 0 | 0 | 0 | 0 | 0 |
| Nocardioides | 0 | 0.5 | 0.4 | 0.8 | 0 | 0 | 0 | 0 | 0 |
| Microvirga | 0 | 0.2 | 1.2 | 0.2 | 0 | 0 | 0 | 0 | 0 |
| Nubsella | 0 | 0 | 0.1 | 0 | 0.3 | 0 | 0.4 | 0.6 | 0.2 |
| Roseomonas | 0 | 0 | 0.4 | 0.7 | 0.1 | 0 | 0.1 | 0.1 | 0.2 |
| f__Rhizobiaceae_83e49f4390f396afb34415853f5470db | 0 | 0 | 0.5 | 0.5 | 0 | 0 | 0.1 | 0.2 | 0.2 |
| Remaining taxa (743) | 5.3 | 50.7 | 33 | 18.6 | 4.2 | 0.9 | 4.8 | 2.6 | 3.3 |

Figure S9: Heatmap showing relative abundance of top 80 genera identified from samples

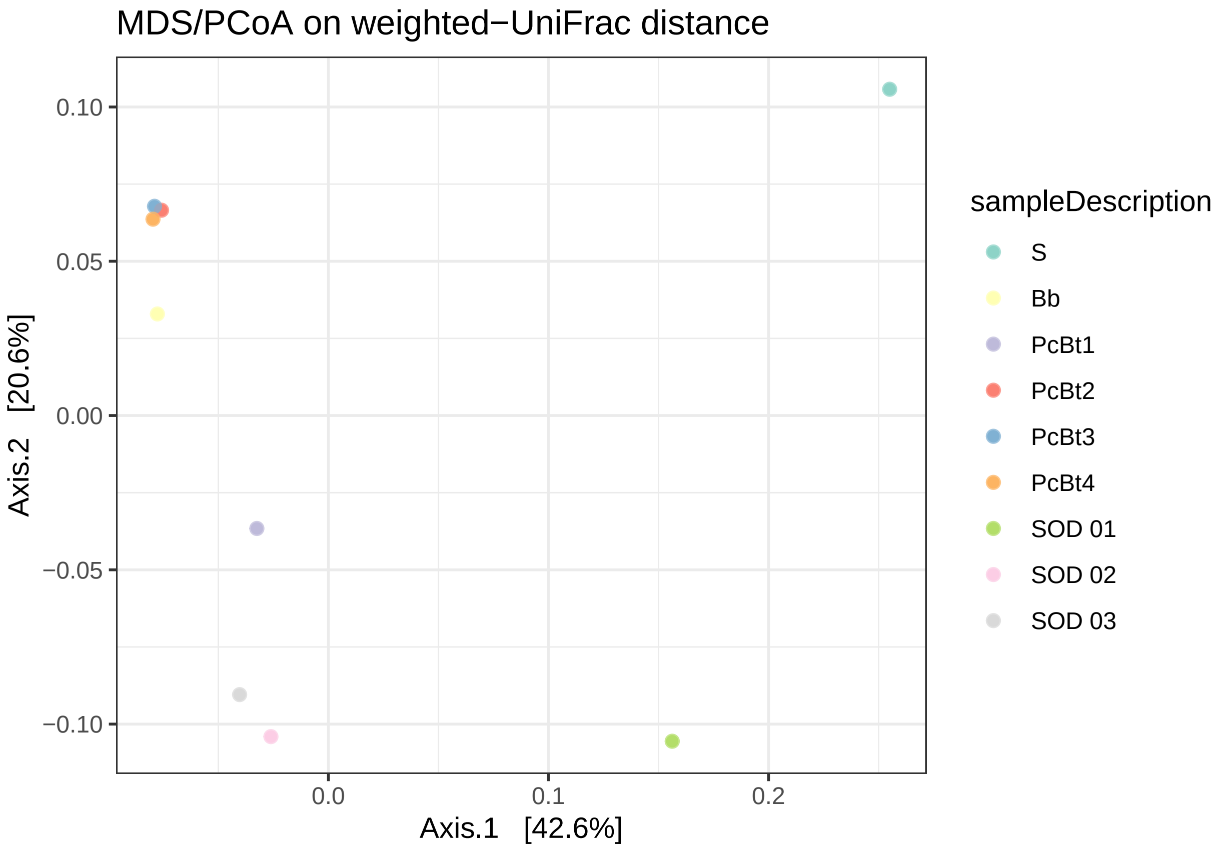


Figure S10: Principal coordinate analysis (multidimensional scaling) of distance matrix using weighted UniFrac metrics. Coordinates for axis 1 and 2 are showin in plot.

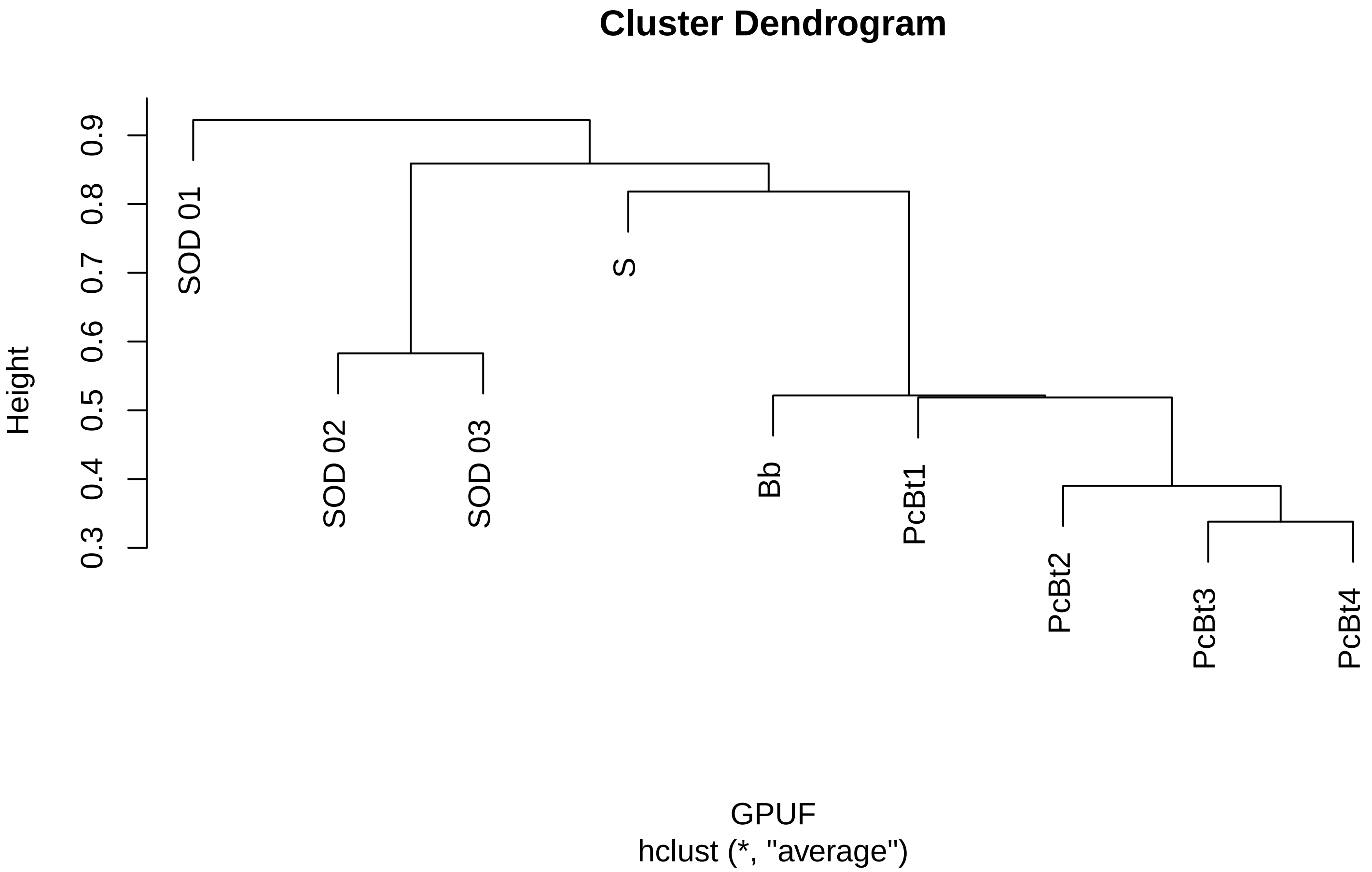


Figure S11: Hierarchical Clustering using UniFrac dissimilarity and UPGMA method.

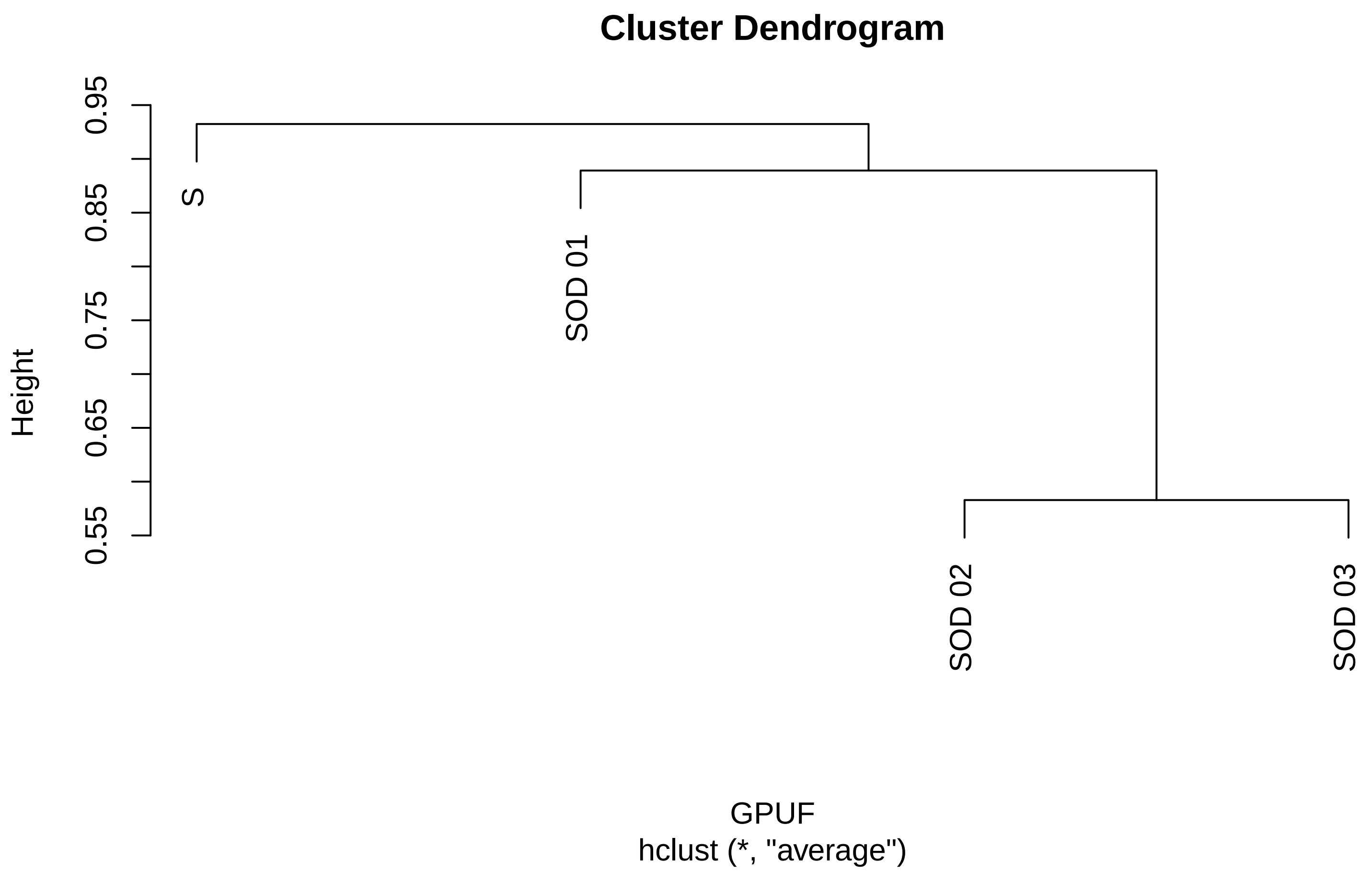


Figure S12: Hierarchical Clustering using UniFrac dissimilarity and UPGMA method of Group A sample group in the present study

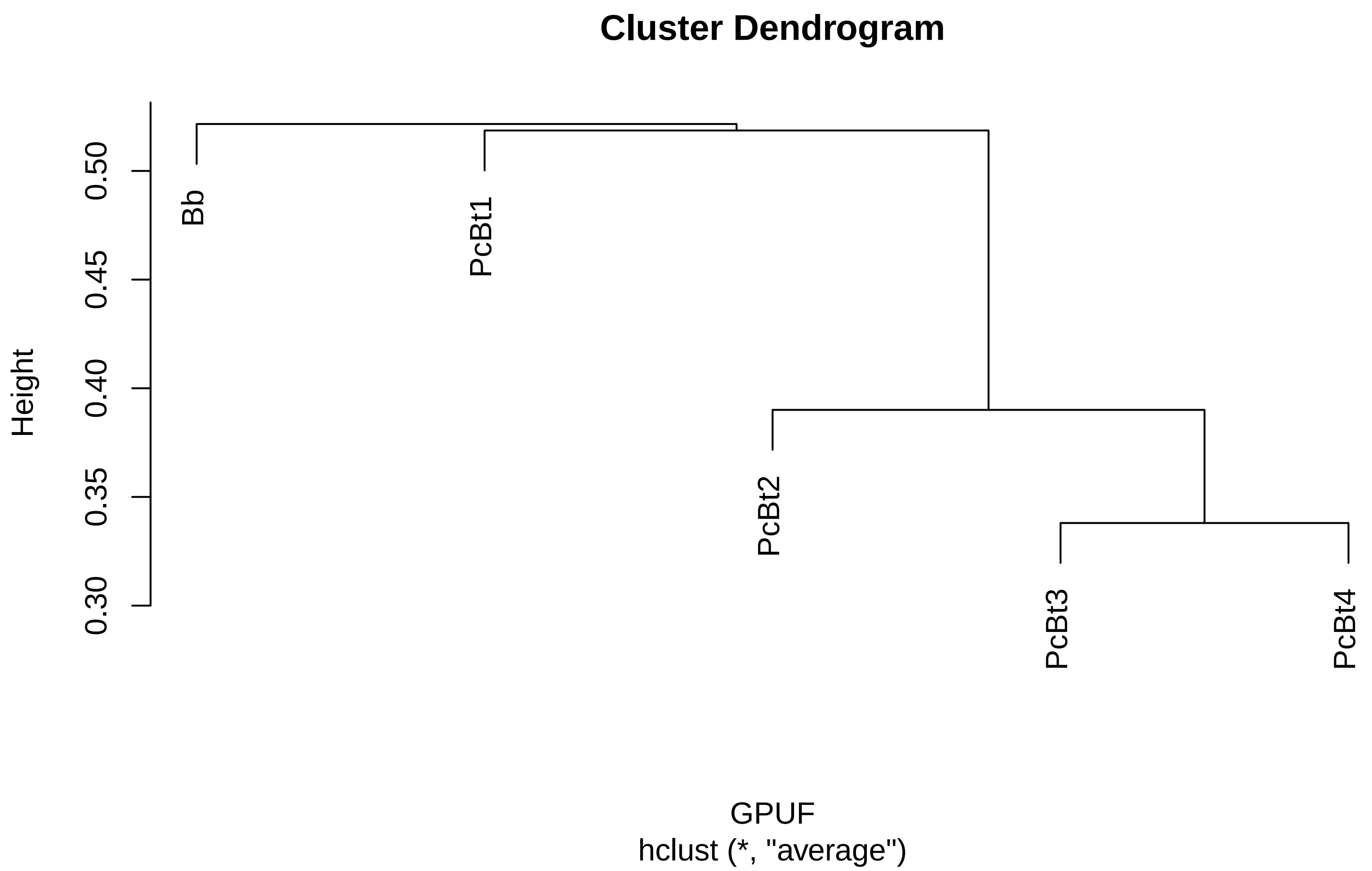


Figure S13: Hierarchical Clustering using UniFrac dissimilarity and UPGMA method of Group B sample group in the present study

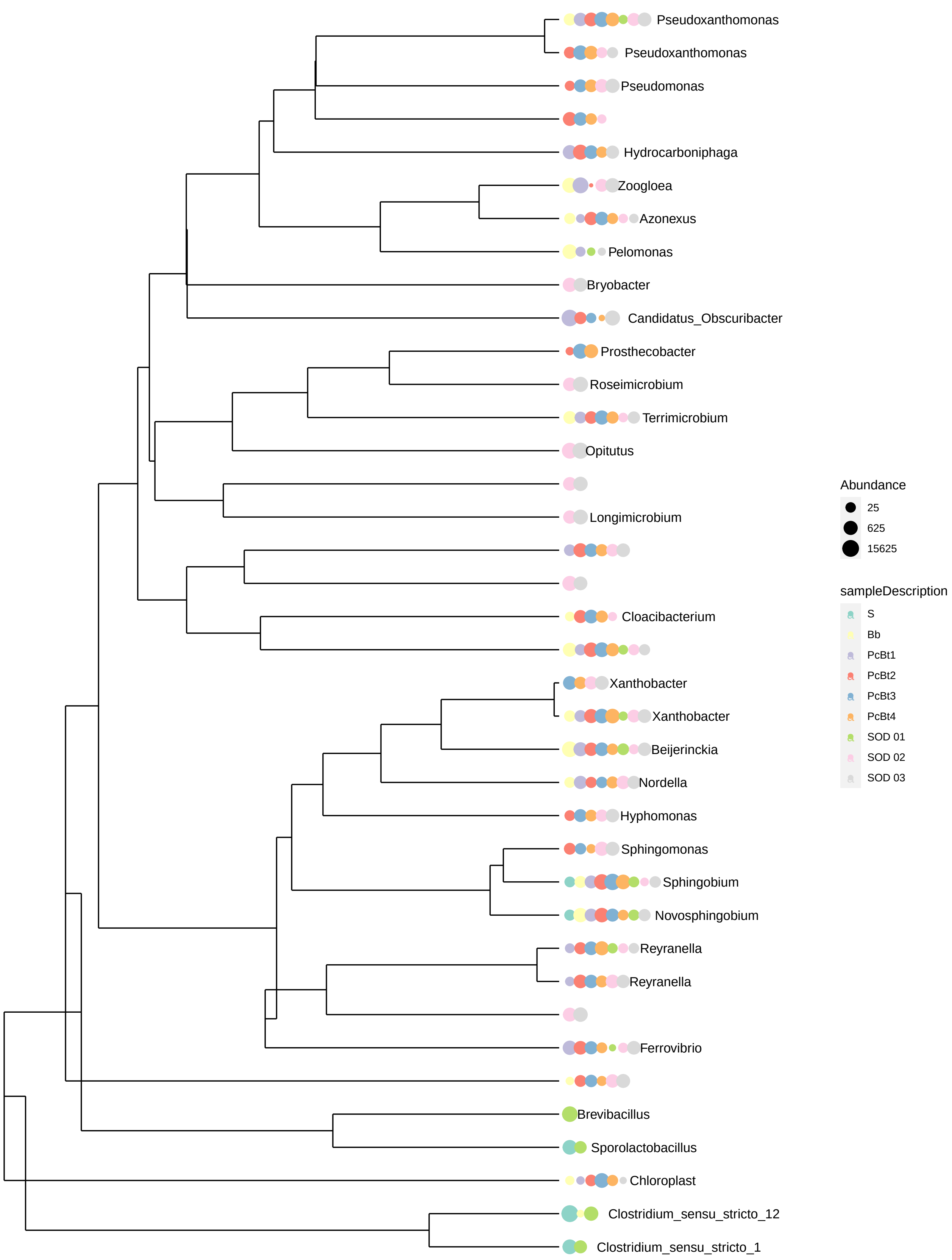


Figure S14: Phylogenetic tree using alignment and UPGMA method done in muscle64.

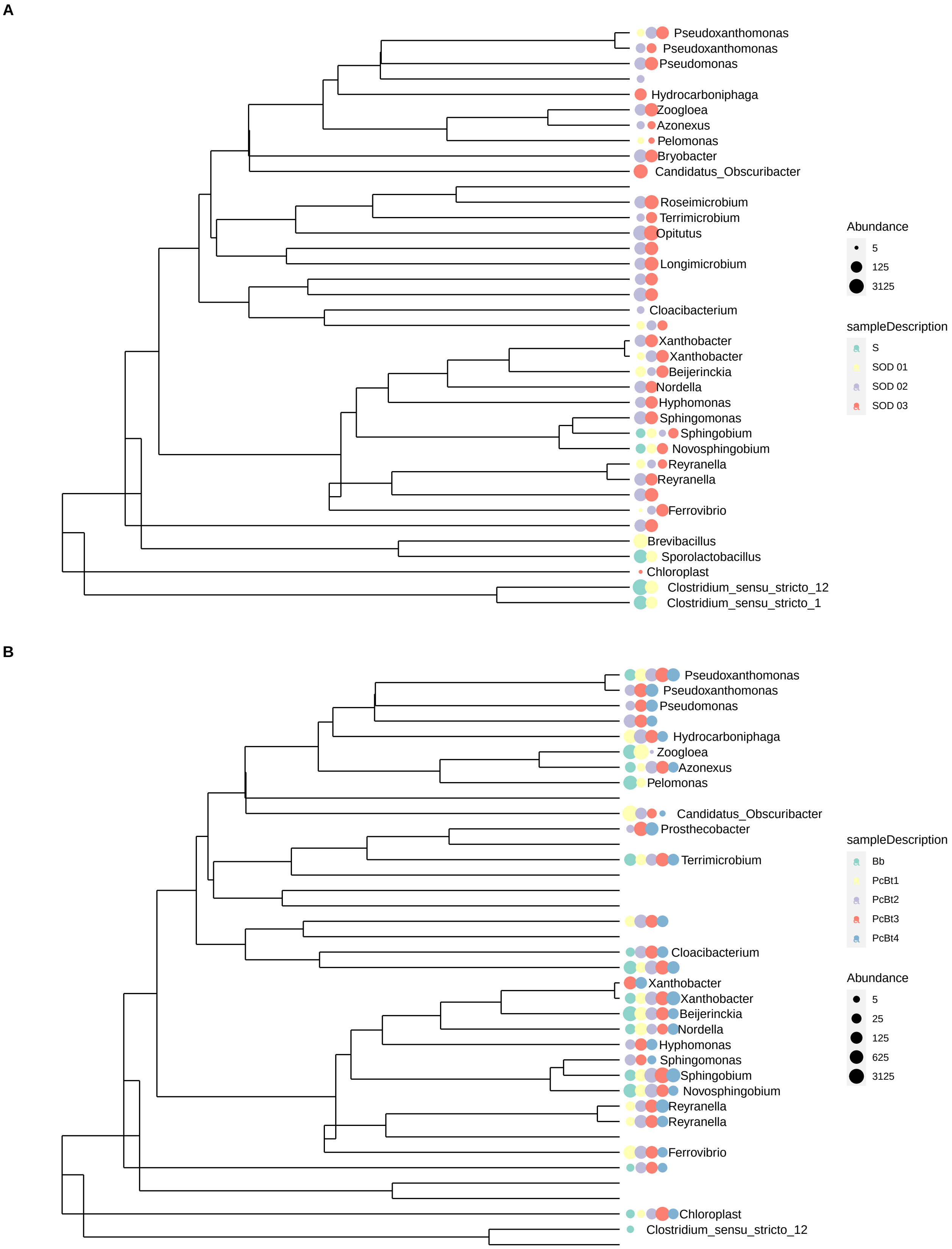


Figure S15: Phylogenetic tree using alignment and UPGMA method done in muscle64 of two sample groups in the present study

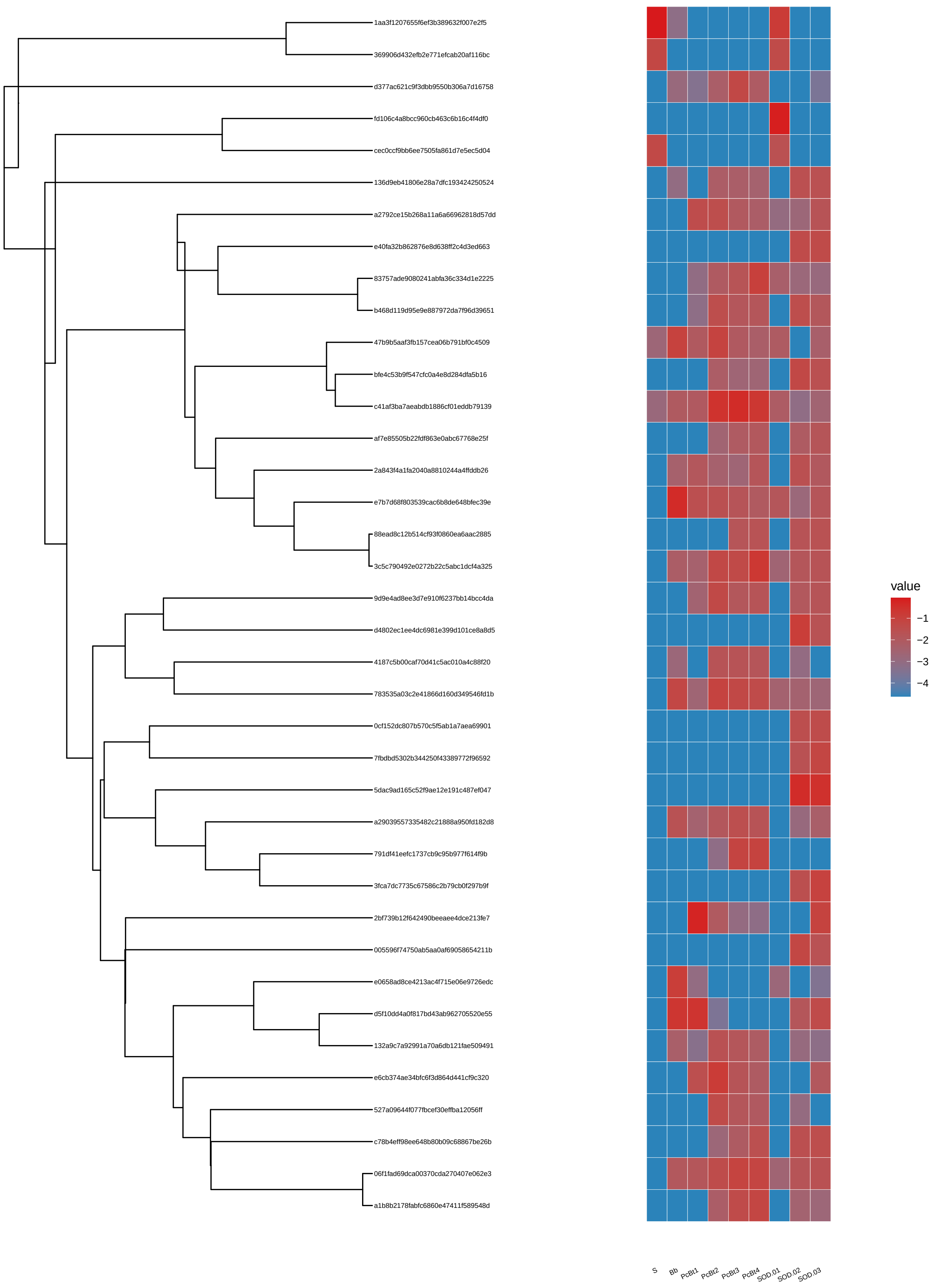


Figure S16: Combined heat map and phylogenetic tree of ASVs with => 1000 total reads (n = 38) from samples.

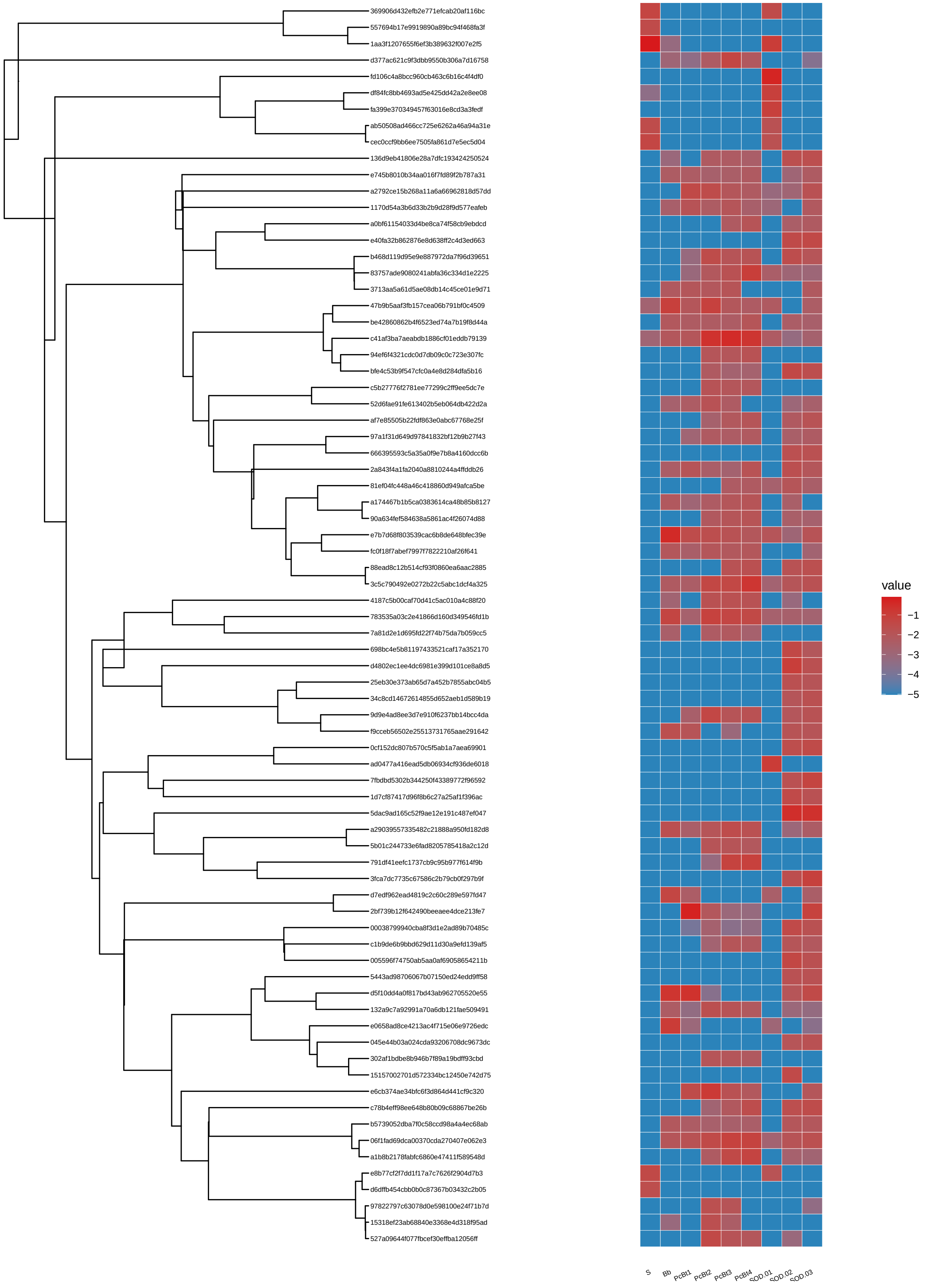

Figure S17: Combined heat map and phylogenetic tree of ASVs with => 500 total reads(n = 76) from samples.
